# Co-Administration of Soy Isoflavones and Vitamin D in Management of Irritable Bowel Disease

**DOI:** 10.1371/journal.pone.0158545

**Published:** 2016-08-04

**Authors:** Mahsa Jalili, Azita Hekmatdoost, Homayoon Vahedi, Hossein Poustchi, Behnam Khademi, Mohsen Saadi, Maryam Zemestani, Leila Janani

**Affiliations:** 1 Department of Biology, Norwegian University of Science and Technology (NTNU), Trondheim, Norway; 2 National Nutrition and Food Technology Research Institute, Faculty of Nutrition and Food Technology, Shahid Beheshti University of Medical Sciences, Tehran, Iran; 3 Digestive disease research center (DDRC), Digestive Disease Research Institute, Tehran, Iran; 4 Liver and pancreatic biliary research group, Digestive Disease Research Institute, Tehran, Iran; 5 Students’ Research Committee, Faculty of Nutrition and Food Technology, Shahid Beheshti University of Medical Sciences, Tehran, Iran; 6 Students’ Research Committee, Faculty of Nutrition, Tabriz University of Medical Sciences, Tabriz, Iran; 7 Department of Biostatistics, School of Public Health, Iran University of Medical Sciences, Tehran, Iran; University Hospital Llandough, UNITED KINGDOM

## Abstract

**Background and Aims:**

The substantial characteristics of Irritable Bowel Syndrome (IBS) are associated with estrogens in women. Both soy isoflavones and vitamin D can modulate estrogen receptors in the colonic smooth muscles. The aim of this study was to investigate the effects of soy isoflavones, vitamin D and their probable interactions in women with IBS.

**Methods:**

In a factorial blinded randomized clinical trial, 100 women with IBS (age:18-75yr, were randomly assigned in 4 arms to receive either placebo of vitamin D and placebo of soy isoflavones (P+P), or placebo of vitamin D and soy isoflavones (P+S), or vitamin D and placebo of soy isoflavones (D+P), or vitamin D and soy isoflavones (D+S) for 6 weeks. Dosage of soy isoflavone was 2 capsules of 20 mg soy isoflavones per day, and dosage of vitamin D was one pearl of 50’000 IU biweekly. The clinical outcomes were IBS symptoms severity scores (IBS-SSS), disease- specific quality of life (IBS-QOL) and total score (IBS-TS) that evaluated at weeks 0, 6, and 10, and compared to each other.

**Results:**

IBS-TS improved significantly in both S+P and D+P groups (p- value = 0.004, 0.015). The interaction effect of soy isoflavones and vitamin D on IBS-TS was significant (p<0.05). The interaction effect of soy isoflavones with vitamin D and the main effect of vitamin D on IBS-SSS were not statistically significant, whereas IBS-SSS decreased significantly in S+P and D+P groups (p-value = 0.001, 0.047 respectively).

**Conclusion:**

Our results indicate that co-administration of soy isoflavones with vitamin D did not improve the IBS- SSS and IBS- QOL; however, it improved the IBS-TS.

**Trial Registration:**

Clinical Trials.gov NCT02026518

## Introduction

Irritable bowel syndrome (IBS) is the most common gastrointestinal problem, which affects 7% to 21% of the general population [1]. Although the prevalence of this syndrome is high, there is no proven treatment for it, and current treatments can negatively impact quality of life [2–4]. The probable mechanisms of IBS include alteration of intestinal permeability, visceral motility and hypersensitivity that can be triggered after an emotional stress [5]. There is a mutual relationship between psychological and somatic risk factors that induce the lifelong symptoms of functional bowel disorders [6]. Hectic lifestyle contributes to high risk of IBS after infections [7] and anxious people experience more frequent and worse symptoms of IBS [8], so that anxiety, stress and emotional illness are associated with increased intestinal permeability and hypersensitivity [5].

Chronic visceral pain is one of the substantial characteristics of IBS that is associated with estrogens activity in women [9]. Women with IBS report more frequent functional bowel disorders during their menstrual period [10]. Reduction of estrogens can affect hypersensitivity and gut permeability of women with IBS [11] that has been shown in experimental Rat model of IBS [12]. Higher severity of symptoms in IBS is correlated with more gut permeability [13]. In the other hand, estrogen receptors type β (ERβ) are expressed predominantly in colon and can be activated by estrogen like compounds such as isoflavones and reduce the gut hypersensitivity [12]. Diadzein, glycetin and genistein are soy isoflavones acting as 17- β estradiol with high affinity to ERβ [14]. Similarly, 1,25 di-hydroxy vitamin D can act as a modulator of ERs and activate the nuclear signaling of these ERs in the colonic smooth muscles[15]. Up to our knowledge, there is no clinical study to evaluate the effect of co-administration of isoflavones and vitamin D on clinical outcomes in IBS. These findings plus a recent report of improvement of IBS symptoms by high-dose supplementation of vitamin D[15] encouraged us to design a randomized clinical trial to evaluate the effects of soy isoflavones and vitamin D supplementation in the management of IBS.

The aim of this study was to investigate the effects of soy isoflavones, vitamin D and their co-administration effects on symptoms severity scores, disease- specific quality of life and total score in women with IBS using a factorial randomized clinical trial design.

## Materials and Methods

### Study design

Women with IBS with age range of 18 to 75 year old and body mass index (BMI) of 18–25 were recruited from the Endoscopy clinic, Shariati Hospital, Tehran, Iran. The ROME III criteria was used for diagnosis of IBS [16]. Inclusion criteria were absence of intestinal organic diseases, intestinal infection, history of colorectal disorders, major intestinal surgery, current use of antibiotics, anti-diarrhea and anti-constipation drugs, non-steroidal anti-inflammatory drugs, metocloperamide, cisaperide, difenoxilate, opium and immune suppressors. The exclusion criteria were use of any type of soy products and/or vitamin D, use of synthetic sweeteners (due to change of intestinal permeability) 2 days before and during the study. Moreover, pregnant and lactating women were not recruited in the study. In addition, patients with history of breast cancer in herself or her mother and sisters were excluded from the study according to their self-reporting. Furthermore, diet therapy, hormone therapy and substantial changes in dietary intakes especially consumption of soy or vitamin D rich foods or supplements during the study, and no desire to continue participation in the study were considered as exclusion criteria.

All patients with eligibility criteria started the study in 2013. The study protocol was approved by Ethics committee of National Nutrition and food Technology Research Institute (NNFTRI) with registeration number of 92-10-10-459. Before starting the intervention, all participants were explained about the intervention, their rights and objectives of the study and they have signed the written informed consent after acceptance to participate in the study. The participants were randomly assigned to receive either placebo of vitamin D and placebo of soy isoflavones (P+P), or placebo of vitamin D and soy isoflavones (P+S), or vitamin D and placebo of soy isoflavones (D+P), or vitamin D and soy isoflavones (D+S) for 6 weeks. We produced 25 equaled size blocks (size = 4) for allocation of patients to 4 different intervention groups. Randomization was not exposed to those conducting the study and was provided in sealed opaque envelopes with successive numbers.

The soy isoflavones capsules (21 century co., USA) contained 10 mg of diadzein, 8.5 mg of genstein and 1.5 mg of glycetin that was taken twice a day. The vitamin D pearls (Zahravi co., Iran) consisted of 50000 IU cholecalciferol that was taken one pearl biweekly. The placebos were in the same shape and color of each homonymous supplement. The placebo of soy isoflavones contained 10 mg corn starch and the placebo of vitamin D contained 10 mg medium chain triglyceride (MCT) oil, and provided by Zahravi Co., Tabriz, Iran. The MCT oil contained 59.4% Caprylic acid, 39.6% Capric acid, 0.7% Caproic acid, 0.2% Lauric acid, and 0.1% Myristic acid.

### Clinical, paraclinical, and dietary intake assessments

The baseline data about personal information, anthropometric data (including weight, height, BMI, waist circumference (WC)) and dietary recalls for 3 days (2 working days and one holiday) was gathered. The dietary recalls were filled by face to face interview and their nutrients content were calculated using dietary calculator software, Nutritionist IV (First data bank). NNFTRI validated list of portion sizes of Iranian cuisine and nutritional composition tables were applied to get accurate data of dietary intake [17].The consumption of soy (foods, supplements) and vitamin D (sun exposure, foods and supplements) was assessed. All participants were asked to fill in a

validated IBS symptoms severity score (IBS-SSS) questionnaire [18] and, a validated IBS quality of life (IBS-QOL) questionnaire [19] at week 0 and week 6. IBS-SSS questionnaire consists of 5 items about the severity of abdominal discomfort, frequency of discomfort, severity of flatulence, satisfaction after defecation and interactive impact of IBS symptoms with everyday life. Data were collected by face to face interview using visual analog scale (VAS) sum of scales were transformed to a 500 scale ranging from 0 to 500. IBS- QOL questionnaire has 34 items including dysphoria (8 items), body image (4 items), health-oriented worries (3 items), sexual related worries (2 items), social behavior (4 items), intervene with every-day activity (7 items) and personal relationship (3 items) [20]. This questionnaire based on 5-choice scale (0–4) and the summed total score was transformed to a 100 scale ranging from 0 (poor QOL) to 100 (maximum QOL); the valid Persian format of original questionnaire was used in this study [19]. Additionally, total score of IBS was measured by a VAS of 100 mm scale to assess the total impact of IBS symptoms on the quality of life at week 0, 6 and 10 (follow-up period) [21]. Weekly follow-up was done by phone call and the probable adverse effects of supplements were asked at each call. Tablet counts were used to assess adherence to treatment and compliance of study participants. The total score of IBS was assessed at week 10 to evaluate the effect of intervention after cessation of supplementation.

The primary outcome measure was score of IBS-SSS questionnaire and the secondary outcome measures were IBS- QOL score and total score.

Based on the result of the study by Ligaarden et al.[22], we used G-Power software to calculate sample size for this study. Considering independent t-test for comparison of means of IBS Score between vitamin D group versus placebo and Soy flavonoid group versus placebo and targeting the significance level of the test at 0.05, twenty one subjects in each group would be enough for detecting 1.6 difference in means of two groups with study power of 80% (S1 = 1.88 and S2 = 1.77). This sample size would be sufficient to detect an interaction effect as large as twice of main effects. Considering 15% loss to follow up, we allocated 25 patients for each group.

### Statistical analysis

Quantitative data were presented as mean (SD) and qualitative data were reported as frequency and percentage. Analysis of variance (ANOVA), chi-square test and fisher’s exact test, were used to compare baseline factors between groups. General linear models (Two-way ANOVA) were used to analyze the main effects and interaction effect of two interventions. The model-based adjusted means (SE) were reported for this type of analysis. Data were analyzed using SPSS software version 20 (Released 2011. IBM SPSS Statistics for Windows, Version 20.0. Armonk, NY: IBM Corp.). P-values < 0.05 were considered statistically significant. Data analyzed by intention-to-treat (ITT) approach. Overall 10% of data were missing. We used regression models for imputing missing values. For reporting results of analysis, we used a table format which introduced by Wang et al [23] for factorial designs.

## Results

**Fig 1:**
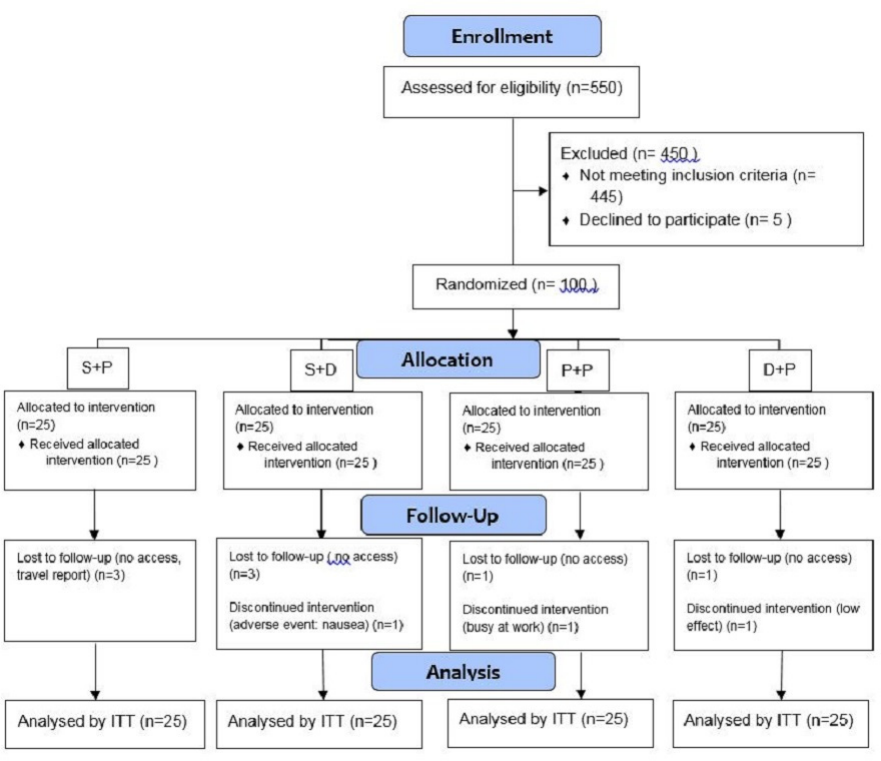
Flow Diagram of the study.

One hundred women with IBS participated in this clinical trial and were assigned to four groups randomly: P+ P group (n = 25), S+P group (n = 25), D+ P group (n = 25) and S+ D group (n = 25). All data on participants’ entrance and retention in the groups have been shown in *Fig 1*. The baseline demographic data of four groups were not significantly different (Table 1). There were no significant differences in dietary intakes among four groups (Results were not shown).

**Table 1:** Characteristics of participants in different groups at the baseline.

	Soy + P (n = 25)	Soy + D (n = 25)	P + P (n = 25)	D + P (n = 25)	P-value
Age (yr) mean(SD)	42.96 (11.70)	40.16 (13.46)	39.76 (12.99)	41.32 (12.62)	0.812^1^
Weight (kg) mean(SD)	71.94 (10.67)	65.60 (14.86)	65.90 (11.95)	66.40 (10.10)	0.204^1^
Height (cm) mean(SD)	160.40 (5.24)	157.54 (5.62)	161.08 (6.09)	159.71 (5.60)	0.130^1^
Waist circumference (cm) mean(SD)	90.50 (9.81)	87.06 (13.95)	87.66 (11.06)	89.00 (11.32)	0.544^1^
BMI (kg/m^2^) mean(SD)	28.08 (4.82)	26.49 (5.87)	25.35 (3.93)	26.04 (4.15)	0.225^1^
Serum vitamin D (ng/ml) mean(SD)	20.87 (8.34)	20.92 (8.32)	21.23 (8.45)	21.10 (8.23)	0.981^1^
Subtypes N (%)					
IBS-C	19 (59.4)	12 (46.2)	22 (62.9)	21 (65.6)	0.374^3^
IBS-D	5 (15.6)	3 (11.5)	4 (11.4)	3 (9.4)	
IBS-M	4 (12.5)	8 (30.8)	7 (20)	5 (15.6)	
IBS-U	4 (12.5)	3 (11.5)	2 (5.7)	2 (6.3)	
IBS-SSS mean (SD)	240.00 (7.1)	240.64 (110.56)	250.56 (120.36)	250.56 (90.60)	0.964^1^
IBS-QOL mean (SD)	64.48 (27.85)	58.48 (31.04)	46.32 (30.06)	58.40 (31.41)	0.195^1^
Total score mean (SD)	21.60 (15.92)	23.80 (13.48)	22.40 (15.55)	20.40 (15.13)	0.879^1^
Smoking N (%)	2 (8.00)	3(12.00)	2 (8.00)	0 (0.00)	0.508^2^
Menopause N (%)	10(40.00)	8 (32.00)	8(32.00)	8(32.00)	0.911^3^

1- Statistical significance test was done by ANOVA.

2- Statistical significance test was done by Fisher’s exact test.

3- Statistical significance test was done by Pearson chi-square.

SD: Standard deviation; Yr: year; Kg: kilogram; M: meter; cm: centimeter; IBS-C: Constipation; IBS-D: Diarrhea; IBS-M: Mixed; IBS-U: Unsubtyped.

### Primary outcome

**Fig 2:**
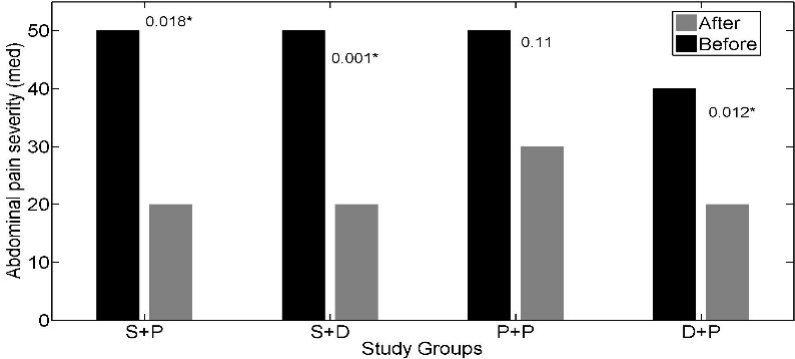
The effects of soy isoflavones and/or vitamin D supplementation on abdominal pain severity after 6 weeks; results are adjusted for the baseline values and IBS subtype. S+P: soy isoflavones and placebo of vitamin D group, S+D: soy isoflavones and vitamin D group, P+P: placebo of soy isoflavones and placebo of vitamin D group, D+P: vitamin D and placebo of soy isoflavones group.* pso of v.

**Fig 3:**
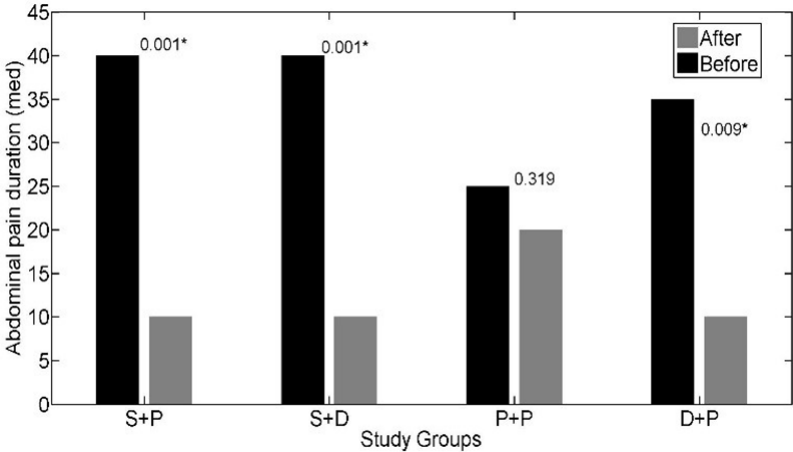
The effects of soy isoflavones and/or vitamin D supplementation on abdominal pain duration after 6 weeks; results are adjusted for the baseline values and IBS subtype. S+P: soy isoflavones and placebo of vitamin D group, S+D: soy isoflavones and vitamin D group, P+P: placebo of soy isoflavones and placebo of vitamin D group, D+P: vitamin D and placebo of soy isoflavones group.* pso of v.

**Fig 4:**
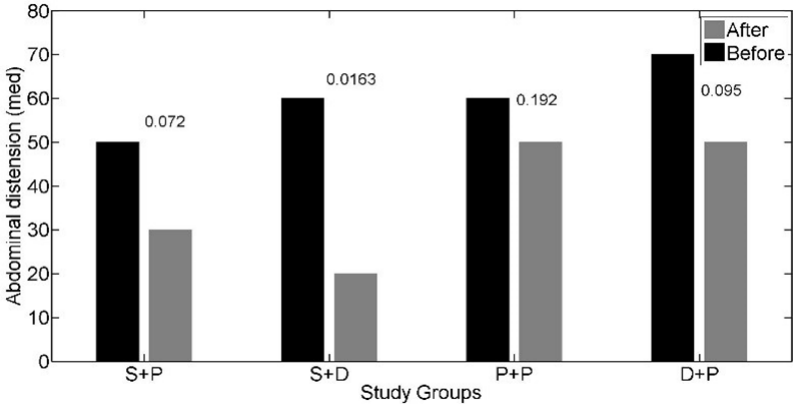
The effects of soy isoflavones and/or vitamin D supplementation on abdominal distension after 6 weeks; results are adjusted for the baseline values and IBS subtype. S+P: soy isoflavones and placebo of vitamin D group, S+D: soy isoflavones and vitamin D group, P+P: placebo of soy isoflavones and placebo of vitamin D group, D+P: vitamin D and placebo of soy isoflavones group.* pso of v.

**Fig 5:**
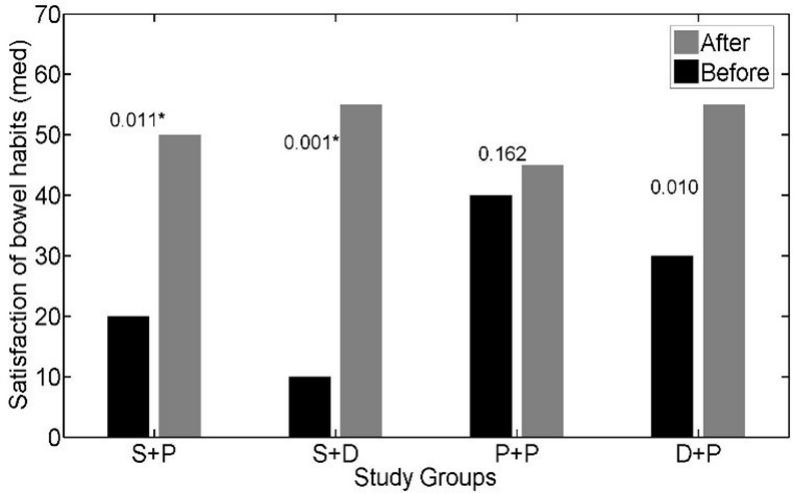
The effects of soy isoflavones and/or vitamin D supplementation on satisfaction of bowel habits after 6 weeks; results are adjusted for the baseline values and IBS subtype. S+P: soy isoflavones and placebo of vitamin D group, S+D: soy isoflavones and vitamin D group, P+P: placebo of soy isoflavones and placebo of vitamin D group, D+P: vitamin D and placebo of soy isoflavones group.* pso of v.

**Fig 6:**
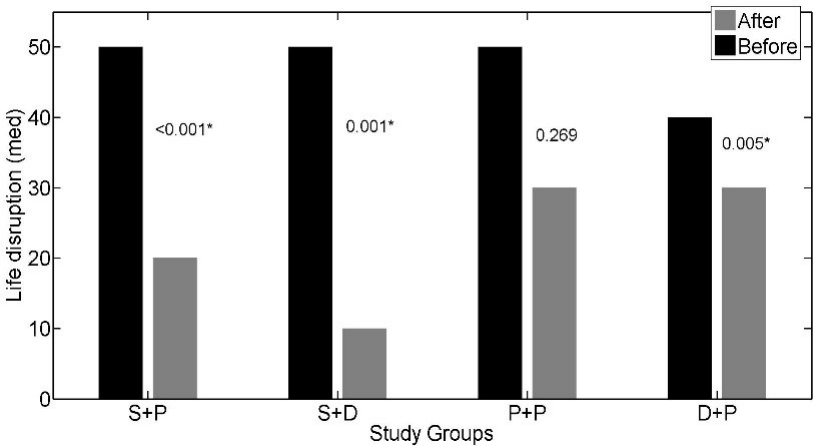
The effects of soy isoflavones and/or vitamin D supplementation on life disruption after 6 weeks; results are adjusted for the baseline values and IBS subtype. S+P: soy isoflavones and placebo of vitamin D group, S+D: soy isoflavones and vitamin D group, P+P: placebo of soy isoflavones and placebo of vitamin D group, D+P: vitamin D and placebo of soy isoflavones group.* pso of v

The interaction effect of soy isoflavones with vitamin D was not statistically significant for IBS-SSS (p = 0.197); so, we could discuss about main effects. The main effects of soy isoflavones and vitamin D on the IBS- SSS were statistically significant (p = 0.001, p = 0.047 respectively). The IBS- SSS was significantly lower in patients who received soy isoflavones versus patients who did not receive it (adjusted mean = 12.93 versus adjusted mean = 17.83). Adjusted mean of IBS- SSS was lower in patients who received vitamin D in comparison to patients who did not receive vitamin D (adjusted mean = 13.59 versus adjusted mean = 16.64) (Table 2). The effects of soy isoflavones and/or vitamin D supplementation on the abdominal pain severity (*Fig 2*), abdominal pain duration (*Fig 3*), abdominal distension (*Fig 4*), satisfaction of bowel habits (*Fig 5*), life disruption (*Fig 6*) after 6 weeks was significantly different among the study groups.

**Table 2:** The effects of soy isoflavones and/or vitamin D supplementation on the IBS- SSS after 6 weeks.

		Vitamin D adjusted mean ^1^(SE)		Total
		No	Yes	
Soy isoflavones adjusted mean^1^ (SE)	No	200.36(10.54)	150.31(10.51)	170.83(10.08)
	Yes	120.91(10.53)	110.86(10.51)	120.93(10.08)
Total		160.64(10.09)	130.59(10.09)	
Soy isoflavones main effect		F = 8.15 (df = 1), P-value = 0.001**		
Vitamin D main effect		F = 0.89 (df = 1), P-value = 0.047*		
Interaction effect		F = 3.79 (df = 1), P-value = 0.195		

1- Adjusted for IBS- QOL and IBS- SSS baseline values,

2- * p-value<0.05;

3- ** p-value<0.001

### Secondary outcomes

The IBS- QOL questionnaires were filled in by all participants at week 0 and week 6. The interaction effect of vitamin D and soy isoflavones on the QOL scores was not statistically significant (p = 0.675); so, we could interpret the main effects of soy isoflavones and vitamin D.Soy isoflavones improved the total score of QOL significantly (adjusted mean for patients who were received soy isoflavones was 34.55 while adjusted mean for patients who were not received it was 44.96, p- value = 0.004). Also, the vitamin D supplementation significantly improved the IBS- QOL scores (The adjusted mean of IBS- QOL score was 35.48 for patients who were received vitamin D, while the adjusted mean for patients who were not received it was 44.04, p- value = 0.015) (Table 3).

**Table 3:** The effects of soy isoflavones or/ and vitamin D on IBS- QOL after 6 weeks.

		Vitamin D adjusted mean^1^(SE)		Total
		No	Yes	
Soy isoflavones adjusted mean^1^ (SE)	No	49.98 (3.51)	39.95 (3.45)	44.96 (2.46)
	Yes	38.09 (3.48)	31.01(3.45)	34.55 (2.46)
Total		44.04 (2.44)	35.48 (2.44)	
Soy isoflavones main effect		F = 8.88 (df = 1), P-value = 0.004*		
Vitamin D main effect		F = 6.12 (df = 1), P-value = 0.015*		
Interaction effect		F = 0.177 (df = 1), P-value = 0.675		

1- Adjusted for IBS- QOL baseline value,

* p-value<0.05

The interaction effect of soy isoflavones and vitamin D on the total score of IBS was significant (p <0.001) (Table 4); so, the overall effects of soy isoflavones and vitamin D on the total score could not be interpreted. However, we assessed the effect of vitamin D on total score in placebo and soy isoflavones groups separately. The effect of vitamin D on the total score was statistically significant in placebo group (the adjusted mean of total score was 68.60 for patients who were received vitamin D while the adjusted mean for placebo group was 29.28, p-value < 0.001).There was not any statistically significant difference between vitamin D and placebo groups in patients who received soy isoflavones in the case of total score (Table 5).

**Table 4:** The effects of soy isoflavones or/ and vitamin D on the total score of IBS after 6 weeks.

		Vitamin D adjusted mean^1^(SE)		Total
		No	Yes	
Soy isoflavones adjusted mean^1^ (SE)	No	29.27(4.42)	69.37(4.35)	49.32(3.10)
	Yes	70.63(4.38)	72.02(4.35)	71.32(3.10)
Total		49.95(3.07)	70.69(3.07)	
Soy isoflavones main effect		F = 24.85 (df = 1), P-value = <0.001**		
Vitamin D main effect		F = 22.76 (df = 1), P-value = <0.001**		
Interaction effect		F = 19.41 (df = 1), P-value = <0.001**		

1- Adjusted for IBS- QOL and total score baseline values

** P-value<0.001

**Table 5:** Estimation of effect of vitamin D on total score in Placebo and Soy isoflavones groups separately.

	Placebo Adjusted mean^1^(SE)	Vitamin D Adjusted mean^1^(SE)	p-value
Placebo (n = 50)	29.28 (4.46)	68.60 (4.46)	<0.001^**^
Soy isoflavones (n = 50)	71.24 (4.34)	72.17 (4.34)	0.882

1- Adjusted for IBS- QOL and total score baseline values

** P-value<0.001

## Discussion

To our knowledge, this study is the first 2× 2 factorial randomized clinical trial that evaluated the effects of soy isoflavones and vitamin D supplementation on clinical manifestations, quality of life and total score in women with IBS. Our findings have revealed that co-administration of soy isoflavones with vitamin D could not improve the IBS- SSS, and IBS- QOL scores, although each treatment reduced significantly the symptoms severity and quality of life. The interaction effect of soy isoflavones and vitamin D on IBS total score was significant, which indicates the synergistic effect of these bioactive metabolites on the total score of IBS.

The total score of IBS was reduced as the interaction effect of soy isoflavones with vitamin D; so, the synergistic effect of these treatments could be possible. The results of follow up total score at week 10 was similar, which indicates that even after cessation of supplements, the beneficial effects of bioactive compounds in suppression of abdominal pain and flatulence was observed. There is no clinical trial evaluating the effects of soy products or vitamin D on clinical manifestation of IBS. The active form of vitamin D can modulate ERs and activate the nuclear signaling of them in the colonic smooth muscles[24]. In vitro studies have shown that both soy phytoestrogens and vitamin D can strengthen each other to bind ERs in smooth muscle cells and modulate the expression of ER proteins[24]. Besides the impact of these nutrients on the specific receptors in colonic tissue, they may affect the psychological items of IBS and soothe the chronic visceral pain [25]. Moreover, vitamin D is an essential nutrient in optimal homeostasis of intestinal mucosal barrier and vitamin D deficiency is related to more severe clinical features of IBS [26].

This study has some limitations: the study was done according to our planned protocol with a good power in sample size estimation; however, the results suggest that we might reach to a better results if the sample size was higher.Another limitation of this study was that judgment of QOL outcomes in randomized clinical trials is sophisticated because there are different specific domains for each QOL item and they are associated with severity of disease, emotional stress, mood and comorbidities [27], however, even small differences in QOL scores can indicate a critical clinical variations [28]. Furthermore, randomization in equal block size of four, although helps to ensure equal group size, may hinder the effectiveness of allocation concealment.

This study has several strengths; one of the strengths of this study is its factorial design, which is an efficient analytical method of concurrently evaluating two independent treatments and finally, application of a disease- specific SSS and QOL questionnaires that demonstrate robust and reliable data on the severity scores and psychometric properties is strength of this study. We conducted a prior study to investigate the effects of soy isoflavones on these clinical outcomes[29]. In this study, we added vitamin D to evaluate their co-administration effect.

In conclusion, the total score of IBS improved significantly through the interaction effect of soy isoflavones and vitamin D that can suggest the synergistic effect of these nutrients on intestinal hypersensitivity. Our findings did not support the synergistic effects of co-administration of soy isoflavones and vitamin D on the IBS- SSS and IBS- QOL, although the soy isoflavones or vitamin D alone reduced the IBS-SSS and IBS- QOL significantly. Our findings provide some clinical evidence for effects of soy isoflavones and vitamin D on IBS symptoms and quality of life. Further clinical trialswith longer follow up and higher sample size are needed to determine the possible effects of co-administration of these supplements on clinical outcomes and management of IBS symptoms clearly.

## Supporting Information

S1 Dataset The raw data. (SAV)

S1 Text The protocol of the study. (DOCX)

S2 Text The Persian protocol of the study. (DOCX)

S3 Text The consort checklist. (DOC)

## Abbreviations

IBS: irritable bowel syndrome
SSS: Symptoms severity score
QOL: quality of life
P: placebo
S: soy isoflavones
D: vitamin D
ER: estrogen receptor
BMI: body mass index
NNFTRI: National Nutrition and food Technology Research Institute
DDRI: Digestive Diseases Research Institute
TUMS: Tehran University of Medical Sciences
MCT: medium chain triglyceride
WC: waist circumference
VAS: visual analog scale
SD: standard deviation
ANOVA: Analysis of variance
ITT: intention-to-treat
