# Supplementary material for: Co-Administration of Soy Isoflavones and Vitamin D in Management of Irritable Bowel Disease
Source: PLoS One. 2016 Aug 4;11(8):e0158545. doi: 10.1371/journal.pone.0158545 (PMC4973900; doi:10.1371/journal.pone.0158545)
Supplement: S1 Text — (DOCX) [file pone.0158545.s002.docx]

**Title:** Soy isoflavones and cholecalciferol for treatment of irritable bowel syndrome: study protocol for a 2× 2 factorial, randomized controlled trial

**Abstract**

**Background**

Irritable bowel syndrome is a common functional gastrointestinal disorder especially in women and there are insufficient high quality and effective clinical trials in this field. This study aims to investigate the interaction and main effects of soy isoflavones and cholecalciferol on symptoms severity, quality of life, total score, and inflammatory markers and gut hyperpermeability in women with IBS.

**Methods/ design**

This study is a factorial randomized controlled trial with 120 eligible patients with IBS that randomly assigned to 4 groups to take soy isoflavones, cholecalciferol or both or none of them in each group for 6 weeks. The follow up session will be at week 10. The primary outcome is symptoms severity and the secondary outcomes are quality of life, total score of disorder, levels of inflammatory markers in serum and blood cells, and the activity of fecal serine protease. The adverse events will be recorded during all sessions of this trial. This study has been approved by Ethics committee in National Nutrition and food Technology Research Institute (NNFTRI) and Digestive Diseases Research Institute (DDRI) in Tehran University of Medical Sciences (TUMS). The ethics committee code is 050459. The findings of this study will contribute to achieve novel effective and safe treatments.

**Discussion:** Up to our knowledge, it will be the first factorial RCT of the impact of soy isoflavones and vitamin D on symptoms severity, QOL, total score, inflammatory markers and fecal serine protease activity in IBS.

**Trial registration:** NCT02026518, clinical trials.gov

**Key words:** Irritable bowel syndrome, Nutrition, Vitamin D, Soy isoflavones, randomized clinical trial

**Background**

Irritable bowel syndrome (IBS) is functional dyspepsia with an epigastric discomfort ([1](#_ENREF_1)). The pathogenesis of this disorder has not clarified yet ([2](#_ENREF_2)). There is an evidence based relationship between symptoms pattern with menstrual cycle in women ([3](#_ENREF_3)). The hypersensitivity and epigastric pain in women with IBS can be due to low activity periods of ovarian hormones before menses ([4](#_ENREF_4)), so ovarian steroids or similar compounds can delay the onset of pain, functional disorder and colonic hyperpermeability in women with IBS ([5](#_ENREF_5)). Furthermore, the estrogen receptors β (ER-β) are expressed predominantly in intestinal mucosa ([4](#_ENREF_4), [6](#_ENREF_6)) and their activity can be modified by estrogen like compounds. Phytoestrogens such as soy isoflavones are similarly structured to ovarian estrogens and can imitate their functions on intestinal mucosa ([7](#_ENREF_7)). In addition, anti- inflammatory bioactive compounds can soothe the inflammatory process and decline the symptoms severity in IBS. The altered food intake in patients with IBS triggers deficiency of some nutrients that can result in aggravation of pain and hypersensitivity ([8](#_ENREF_8)). The active metabolite of vitamin D can modulate the ERs in intestinal smooth muscles, so it can be effective in treatment of symptoms in IBS ([9](#_ENREF_9)). Intestinal hyperpermeability is one of the possible mechanisms of pathogenesis of IBS that aggravate the pain and flatulence occurrence in patients and luminal serine protease can be a relevant marker of pathology of IBS ([10](#_ENREF_10)). Emotional stress can induce higher protease activity in colon and develop more severe symptoms of IBS ([11](#_ENREF_11)). The Quality of life (QOL) impaired significantly in patients with IBS ([12](#_ENREF_12)). Poor QOL in IBS results in high economic loss due to several days of absence of work ([13](#_ENREF_13)). Suppression of symptoms of gastrointestinal dysfunction can contribute to improve QOL ([14](#_ENREF_14)). Nevertheless the prevalence of IBS is high among gastrointestinal disorders and in a recent paper, the prevalence of IBS has been reported between 10 to 20 % ([15](#_ENREF_15)), there are rare high quality RCTs in this field. Moreover, in accordance with the similar functions of soy isoflavones with vitamin D on intestinal mucosa, we could not find any relevant RCT, so to best of our knowledge, this factorial RCT is the first clinical trial on the interaction effect and the main effects of phytoestrogens and vitamin D on symptoms, QOL and gut permeability in women with IBS.

**Methods/ Design**

**Objectives**

The primary objective of this study is to evaluate the main effects and interaction effect of them on IBS severity scores and collect the preliminary data on feasibility and efficacy of these intervention for future larger RCTs in patients with IBS.

**Design**

This study is a double blinded RCT with factorial design. The study will be conducted in endoscopy clinic, Shariati Hospital, Tehran, Iran. This protocol has been approved by Ethics committee in National Nutrition and food Technology Research Institute (NNFTRI) and Digestive Diseases Research Institute (DDRI) in Tehran University of Medical Sciences (TUMS), Tehran, Iran. Written informed consent will be got from all participants after oral and written explanation about the procedure of the study.

The participants that enter to this study will be selected according to eligibility criteria and will be randomized by computerized block randomization method with block size of 6 and will take the intervention for 6 weeks. The participants will be followed up at week 10 (Fig. 1). The assigned four groups will be placebo of vitamin D and placebo of soy isoflavones (P+P), placebo of vitamin D and soy isoflavones (P+S), vitamin D and placebo of soy isoflavones (D+P), vitamin D and soy isoflavones (D+S). The capsule of soy isoflavones contains 10 mg of diadzein, 8.5 mg of genstein and 1.5 mg of glycetin that will be taken twice per day. The vitamin D pearl consists of 50000 IU cholecalciferol that will be taken one per 15 days.

The control group will be obtained by use of placebo of soy isoflavones or cholecalciferol instead of original pill. The placebo of each treatment is in the same color and size.

**Eligibility criteria**

**Inclusion criteria**

Age 18-75 yrs; patients with IBS according to ROME III criteria ([16](#_ENREF_16)); Body Mass index (BMI) 18-25; no intestinal organic diseases; no intestinal infection; no history of chronic gastrointestinal and colorectal diseases; no intestinal major surgery; no regular use of antibiotics, anti-constipation and anti-diarrhea, immune suppressors, metocloperamide, cisaperide, difenoxilate, opium and non-steroidal anti-inflammatory drugs; no pregnancy and lactation; not athlete or bed rest; no history of breast cancer in herself or her family; no severe psychosis

**Exclusion criteria**

Use of soy isoflavones or vitamin D one year before the study; use of soy milk or soy nuts during study; diet changes during study; use of artificial sweetener 2 days before study; no desire to complete the study; adverse effect of supplement; pregnancy during study

**Outcome Measures**

**Primary outcome**

The severity of symptoms is the primary outcome of this protocol that will be assessed by validated IBS symptoms Severity Score (IBS- SSS) questionnaire at week 0 and week 6. IBS-SSS consists of 5 items on abdominal discomfort, its frequency, flatulence, satisfaction after defecation and interactive impact of IBS on the everyday life. This questionnaire will be filled by interview as VAS and the total score will be between 0 and 500 ([17](#_ENREF_17)).

**Secondary outcomes**

QOL in IBS will be assessed by validated IBS- QOL questionnaire at week 0 and week 6. IBS- QOL questionnaire has 34 items on dysphoria (8 items), body image (4 items), health-oriented worries (3 items), sexual related worries (2 items), social behavior (4 items), intervene with every-day activity (7 items) and personal relationship (3 items) ([18](#_ENREF_18)). Total score of IBS, another secondary outcome will be determined by a VAS between 0 and 100 –mm to evaluate the effect of interventions on total severity of IBS at week 0, week 6 and week 10 (follow up)([17](#_ENREF_17)).

Fecal serine protease enzyme activity is the marker for gut hyperpermeability, as one of the secondary outcomes will be measured by enzyme- linked immunosorbant assay (ELISA) kit after collection of fecal samples, keep them in -80◦ C freezer and preparation by 5 ml Tris buffer and centrifuged in 3000 RCF for 10 min.

Ten ml fasting venous blood samples will be taken from all participants that separated in 3 microtubes: 1- a microtube with EDTA as anti-coagulant will be centrifuged at 3000 RCF for 10 min to separate plasma and whole blood. 2- a microtube without any type of anti-coagulants will be kept for 30 min at room temperature, then will be centrifuged at 3000 RCF for 10 min to separate serum. 3- a microtube with heparin as anti-coagulant will be used to separate PBMC and culture them followed by centrifuging at 2000 RCF for 15 min. Isolated PBMC will be used to compare the amount of Nuclear Factor κβ (NF- κβ) before and after the intervention. All separated samples will be collected quickly in crayotubes and will be transferred to -80◦ C freezer until experiments start. Sera will be recovered to determine the level of serum 25- OH vitamin D and Tumor Necrosis factor- α (TNF- α) at week 0 and week 6.

**Statistical Methods**

**Sample Size**

According the factorial design, we consider required sample size to compare soy isoflavones group versus control group and vitamin D group versus control group, then the maximum predicted value is considered as final required sample size to assess the efficacy of the interaction effect and the main effects. A similar study ([19](#_ENREF_19)) is used to determine the sample size by T test to compare the means of QOL. The probability of type 1 error is 5% and the probability for type 2 error is 20%, so the calculated sample size is 21 persons per each group. The G power software version 3-1-7 is used to calculate sample size. The probability of dropout is considered 15%, so the predicted number increases to 30 for each group and we will require 120 patients with IBS. This number of patients is sufficient to find the interaction effect as 2 times of main effects, although we will need more sample size if we desire to find much smaller scale of differences.

**Statistical Analysis**

All data will be analyzed by SPSS version 20 statistical package. The levels of significance will be considered at P lower than 0.05. Intention to Treat (ITT) will be applied for all primary and secondary outcomes data. Quantitative data will be reported as mean (SD) or (SEM), qualitative data will be reported as frequency (percent). Baseline data will be analyzed by ANOVA, Fisher’s exact test or X^2^ test. Linear regression models will be used to analyze the interaction effect and the main effects.

**Safety**

Any unfavorable reported symptoms will be recorded in specific forms and 4 weeks after cessation of intervention, all the remained will be asked about the compliance of intervention, personal attitude on this study and any adverse events will be reported even in follow up period.

**Discussion**

Up to our knowledge, it will be the first factorial RCT of the impact of soy isoflavones and vitamin D on symptoms severity, QOL, total score, inflammatory markers and fecal serine protease activity in IBS. This study will be investigated to determine the efficacy of two interventions or their any probable synergistic effect on IBS.

IBS-SSS, IBS-QOL and total score will be recorded by face to face interview and similar training sessions will be held for all research team to reduce the information and selection bias and inter-individual variability among the interviewers.

The step by step manual for laboratory analysis will be provided to prevent any random error in long duration of investigations. Furthermore, all analyses will be done at research lab of DDRI to control any systematic error of lab systems and technicians.

The strength points of this study can be the following items: Vitamin D deficiency is prevalent in our country especially in Tehran because of the latitude of this city and the impact of air pollution, Moreover there is enough evidence on the deficiency of vitamin D especially in women with IBS, so we select the women with IBS that reside in Tehran. Data management will be done at -1 week, week 0, week 6 and week 10 by manually checking of data accuracy and relevancy by one the members of research team. Furthermore, the statistician will clean all data after entrance of all data in statistical software and will report any unreal data to data collection members to check again.

The amount of TNF-α and NF- κβ is a critical marker of mild inflammation level in IBS and the effect of intervention can be critical for future RCTs. Fecal serine protease is an acceptable and feasible marker for intestinal permeability that is safe and with the minimum offensive consequence on the intestinal tissues compared to biopsy samples of intestine during colonoscopy ([20](#_ENREF_20)).

This study can contribute to specify the marginal means of soy isoflavones and vitamin D and any interaction effect of them on clinical and biological outcomes in IBS ([21](#_ENREF_21)). The potential possibility of large lost to follow up in most clinical trials on IBS makes this clinical research inexplicit to report any realistic findings while it can be assuring to evaluate the efficacy and safety of the interventions in IBS for future larger population based RCTs.

**List of abbreviations:**

IBS: irritable bowel syndrome, SSS: Severity Scoring System, QOl: quality of life, RCT: randomized clinical trial, TNF-α: Tumor Necrosis Factor- alpha, NF- κβ: Nuclear Factor- Kappa Beta, ELISA: enzyme- linked immunosorbant assay, PBMC: Peripheral blood mononuclear cells, DDRI: Digestive Diseases Research Institute, TUMS: Tehran University of Medical Sciences, SBMU: Shahid Beheshti University of Medical Sciences, EDTA: ethylenediaminetetraacetic acid

**Competing interests:** There is no competing interest.

**Funding:** Shahid Beheshti University of Medical Sciences, Tehran, Iran.

**References**

**1. Talley NJ, Locke GR, 3rd, Lahr BD, Zinsmeister AR, Tougas G, Ligozio G, et al. Functional dyspepsia, delayed gastric emptying, and impaired quality of life. Gut. 2006;55(7):933-9. Epub 2005/12/03.**

**2. Talley NJ, Verlinden M, Jones M. Can symptoms discriminate among those with delayed or normal gastric emptying in dysmotility-like dyspepsia? The American journal of gastroenterology. 2001;96(5):1422-8. Epub 2001/05/26.**

**3. Tack J, Bisschops R. Mechanisms underlying meal-induced symptoms in functional dyspepsia. Gastroenterology. 2004;127(6):1844-7. Epub 2004/12/04.**

**4. Houghton LA, Lea R, Jackson N, Whorwell PJ. The menstrual cycle affects rectal sensitivity in patients with irritable bowel syndrome but not healthy volunteers. Gut. 2002;50(4):471-4. Epub 2002/03/13.**

**5. Moussa L, Bezirard V, Salvador-Cartier C, Bacquie V, Houdeau E, Theodorou V. A new soy germ fermented ingredient displays estrogenic and protease inhibitor activities able to prevent irritable bowel syndrome-like symptoms in stressed female rats. Clinical nutrition (Edinburgh, Scotland). 2013;32(1):51-8. Epub 2012/06/26.**

**6. Ritchie J. Pain from distension of the pelvic colon by inflating a balloon in the irritable colon syndrome. Gut. 1973;14(2):125-32. Epub 1973/02/01.**

**7. Thompson WG, Heaton KW, Smyth GT, Smyth C. Irritable bowel syndrome in general practice: prevalence, characteristics, and referral. Gut. 2000;46(1):78-82. Epub 1999/12/22.**

**8. Williams EA, Nai X, Corfe BM. Dietary intakes in people with irritable bowel syndrome. BMC gastroenterology. 2011;11:9. Epub 2011/02/05.**

**9. Somjen D, Kohen F, Gayer B, Knoll E, Limor R, Baz M, et al. A non-calcemic Vitamin D analog modulates both nuclear and putative membranal estrogen receptors in cultured human vascular smooth muscle cells. The Journal of steroid biochemistry and molecular biology. 2004;89-90(1-5):397-9. Epub 2004/07/01.**

**10. Roka R, Rosztoczy A, Leveque M, Izbeki F, Nagy F, Molnar T, et al. A pilot study of fecal serine-protease activity: a pathophysiologic factor in diarrhea-predominant irritable bowel syndrome. Clinical gastroenterology and hepatology : the official clinical practice journal of the American Gastroenterological Association. 2007;5(5):550-5. Epub 2007/03/06.**

**11. Song SW, Park SJ, Kim SH, Kang SG. Relationship between irritable bowel syndrome, worry and stress in adolescent girls. Journal of Korean medical science. 2012;27(11):1398-404. Epub 2012/11/21.**

**12. Hou X, Chen S, Zhang Y, Sha W, Yu X, Elsawah H, et al. Quality of life in patients with Irritable Bowel Syndrome (IBS), assessed using the IBS-Quality of Life (IBS-QOL) measure after 4 and 8 weeks of treatment with mebeverine hydrochloride or pinaverium bromide: results of an international prospective observational cohort study in Poland, Egypt, Mexico and China. Clinical drug investigation. 2014;34(11):783-93. Epub 2014/09/27.**

**13. Lackner JM, Gudleski GD, Ma CX, Dewanwala A, Naliboff B. Fear of GI symptoms has an important impact on quality of life in patients with moderate-to-severe IBS. The American journal of gastroenterology. 2014;109(11):1815-23. Epub 2014/09/17.**

**14. Spiegel BM, Gralnek IM, Bolus R, Chang L, Dulai GS, Mayer EA, et al. Clinical determinants of health-related quality of life in patients with irritable bowel syndrome. Archives of internal medicine. 2004;164(16):1773-80. Epub 2004/09/15.**

**15. Staudacher HM, Lomer MC, Anderson JL, Barrett JS, Muir JG, Irving PM, et al. Fermentable carbohydrate restriction reduces luminal bifidobacteria and gastrointestinal symptoms in patients with irritable bowel syndrome. The Journal of nutrition. 2012;142(8):1510-8. Epub 2012/06/29.**

**16. Wang X, Luscombe GM, Boyd C, Kellow J, Abraham S. Functional gastrointestinal disorders in eating disorder patients: altered distribution and predictors using ROME III compared to ROME II criteria. World journal of gastroenterology : WJG. 2014;20(43):16293-9. Epub 2014/12/05.**

**17. Sisson G, Ayis S, Sherwood RA, Bjarnason I. Randomised clinical trial: A liquid multi-strain probiotic vs. placebo in the irritable bowel syndrome--a 12 week double-blind study. Alimentary pharmacology & therapeutics. 2014;40(1):51-62. Epub 2014/05/13.**

**18. Gholamrezaei A, Zolfaghari B, Farajzadegan Z, Nemati K, Daghaghzadeh H, Tavakkoli H, et al. Linguistic validation of the Irritable Bowel Syndrome-Quality of Life Questionnaire for Iranian patients. Acta medica Iranica. 2011;49(6):390-5. Epub 2011/08/30.**

**19. Bloedon LT, Jeffcoat AR, Lopaczynski W, Schell MJ, Black TM, Dix KJ, et al. Safety and pharmacokinetics of purified soy isoflavones: single-dose administration to postmenopausal women. The American journal of clinical nutrition. 2002;76(5):1126-37. Epub 2002/10/26.**

**20. Gecse K, Roka R, Ferrier L, Leveque M, Eutamene H, Cartier C, et al. Increased faecal serine protease activity in diarrhoeic IBS patients: a colonic lumenal factor impairing colonic permeability and sensitivity. Gut. 2008;57(5):591-9. Epub 2008/01/16.**

**21. Mdege ND, Brabyn S, Hewitt C, Richardson R, Torgerson DJ. The 2 x 2 cluster randomized controlled factorial trial design is mainly used for efficiency and to explore intervention interactions: a systematic review. Journal of clinical epidemiology. 2014;67(10):1083-92. Epub 2014/07/27.**
