# Supplementary material for: Co-Administration of Soy Isoflavones and Vitamin D in Management of Irritable Bowel Disease
Source: PLoS One. 2016 Aug 4;11(8):e0158545. doi: 10.1371/journal.pone.0158545 (PMC4973900; doi:10.1371/journal.pone.0158545)
Supplement: S2 Text — (DOCX) [file pone.0158545.s003.docx]

# بسمه تعالي

######
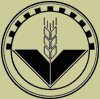


**انستيتو تحقيقات تغذيه‌اي و صنايع غذايي كشور**

##### معاونت پژوهشي

###### **كاربرگ پيشنهاد طرح پژوهشي**

**1- مشخصات كلي طرح**

| عنوان طرح به فارسی: | تاثیر کوله کلسیفرول، ایزوفلاونهای سویا و تجویز توام انها در مقایسه با دارونما بر علایم بالینی، کیفیت زندگی، سیتوکین التهابی TNF-alpha، ظرفیت تام آنتی اکسیدانی سرم TAC و میزان نفوذپذیری روده در بیماران مبتلا به سندروم روده تحریک پذیر | | | |
| --- | --- | --- | --- | --- |
| **عنوان طرح به انگليسي:** | The effects of cholecalciferol, soy isoflavones, and co-administration of them versus placebo on clinical outcomes, quality of life, TNF-alpha inflammatory cytokine, serum total antioxidant capacity and gut permeability in patients with irritable bowel syndrome | | | |
| \| رديف \| نام و نام خانوادگي \| تعلق سازماني \| \| --- \| --- \| --- \| \| 1 \| آزیتا حکمت دوست \| استادیار (MD-PhD علوم تغذیه) \| \| 2 \| مهسا جلیلی \| دانشجوی دکترای تخصصی علوم تغذیه \|   **نام مجري/مجري‌هاي طرح و تعلق سازماني:** | | | | |
| **طول مدت اجراي طرح:** | | **24 ماه** | **تاريخ تقريبي شروع طرح: تابستان1392** | |
| **بودجه مورد نياز انجام طرح تحقيقاتي: (**بودجه‌اي كه مجموعاً از طرف انستيتو به منظور اجراي طرح پرداخت خواهد شد.) | | |  | **100000000ريال** |
| **مجموع نيروي انساني:** | | |  | **ريال** |
| **جمع هزينه‌هاي خدمات (**شامل هزینه های آزمایشات، مسافرت، تکثیر و تایپ و سایر هزینه ها)**، و کالاهای مصرفی:** | | |  | **102025000ريال** |
| **هزينه خريد كالاهاي سرمايه‌اي:** | | |  | **ريال** |
| **نام سازمان/سازمان‌هاي ديگر كه بخشي از بودجه به‌وسيله آن/آنها پرداخت مي‌شود:** مرکز تحقيقات بيماريهاي گوارش و کبد دانشگاه تهران | | |  | **ريال** |
| **نام سازمان/سازمان‌هاي ديگر كه بخشي از خدمات و مواد به‌وسيله آن/آنها تأمين مي‌شود:** مرکز تحقيقات بيماريهاي گوارش و کبد دانشگاه تهران | | |  | **ريال** |
| **محل انجام طرح:** مرکز تحقيقات بيماريهاي گوارش و کبد دانشگاه تهران  **محل انجام آزمایشات:** آزمایشگاه تحصیلات تکمیلی  **کل بودجه مورد نیاز برای انجام طرح:250000000ریال** | | | | |
| **نوع پژوهش:**  **(در صورتیکه طرح دارای دامنه کاربرد دو یا چند بعدی است، به طور همزمان تمامی جنبه ها را مشخص کنید.)** | | **پايه‌اي** ■ **کاربردی** ■ **توسعه ای نظام سلامت (HSR)** | | |
| **در صورت تصویب به عنوان پايان نامه دانشجويي:** | | **مقطع کارشناسی ارشد مقطع دكترا** ■  **نوع مطالعه: کارآزمایی بالینی تصادفی دو سو کور با طرح فاکتوریال** | | |
| **در صورتيكه طرح سفارش مستقيم نهاد اجرايي يا صنعت است نام سفارش‌دهنده ذكر شود:** | | | | |
| **نوع طرح از نظر درجه نوآوري: نوآورانه** ■ **توسعه دانش حاضر تاييد دانش حاضر**  **مصرف‌ كنندگان اصلي نتايج اين پژوهش:** بیماران مبتلا به سندروم روده تحریک پذیر، پژوهشگران حیطه بیماریهای گوارشی  **تعداد مقالات علمي پژوهشي پيش‌بيني شده حاصل از انجام اين طرح:**  **داخلي: 2 نمايه‌شده در ISI و Pubmed: یک** | | | | |

**2- اسامي و مشخصات گروه پژوهش**

**عنوان طرح:** تاثیر کوله کلسیفرول، ایزوفلاونهای سویا و تجویز توام انها در مقایسه با دارونما بر علایم بالینی، کیفیت زندگی، سیتوکین التهابی TNF-alpha، ظرفیت تام آنتی اکسیدانی سرم TAC و میزان نفوذپذیری روده در بیماران مبتلا به سندروم روده تحریک پذیر

^*^مجريان طرح

| **رديف** | **نام و نام خانوادگي** | نشاني الكترونيكي(E-mail) | شماره شناسنامه **صادره از - و کدملی** | تلفن همراه | **رشته/ گرايش تخصصي** | **^**^نوع همكاري** | **امضا** |
| --- | --- | --- | --- | --- | --- | --- | --- |
| 1 | آزیتا حکمت دوست | a_hekmat2000@yahoo.com | 5496 از تهران  0064072551 | 09123065084 | PhD علوم تغذیه | مدیریت عملیات میدانی و مشاوره متدولوژی |  |
| 2 | مهسا جلیلی | Jalili.mah@gmail.com | 6393 از تبریز  1382551967 | 09143147303 | دانشجوی دکترای علوم تغذیه | نگارش پروپوزال، گزارش­های پیشرفت و گزارش پایانی، انجام آزمایشات، طراحی پرسشنامه، مدیریت اطلاعات، ورود اطلاعات و تجزیه و تحلیل آماری |  |

#### همكاران اصلي طرح

| **رديف** | **نام و نام خانوادگي** | نشاني الكترونيكي(E-mail) | تلفن همراه | **رشته/گرايش تخصصي** | **^**^نوع همكاري** | **امضا** |
| --- | --- | --- | --- | --- | --- | --- |
| 1 | همایون واحدی | [homayoon vahedi@gmail.com](https://mail.google.com/mail/u/0/h/pzpdl3i0wrvi/?&v=ct&ct_id=4372d6b09489c88) | 09121095771 | فوق تخصص گوارش | مدیریت عملیات میدانی و مشاوره متدولوژی و تشخیص بیماری |  |
| 2 |  |  |  |  |  |  |

**مشاوران طرح**

| **رديف** | **نام و نام خانوادگي** | نشاني الكترونيكي(E-mail) | تلفن همراه | **رشته/گرايش تخصصي** | **^**^نوع همكاري** | **امضا** |
| --- | --- | --- | --- | --- | --- | --- |
| 1 |  |  |  |  |  |  |
| 2 |  |  |  |  |  |  |

***در صورتي كه محل كار مجري خارج از انستيتو و دانشكده باشد, كارنامه فعاليتهاي پژوهشي (CV) ضميمه گردد.**

**** به عنوان مثال: مشاوره آماري، انجام تجزيه تحليل‌هاي آماري، مشاوره متدولوژي، مشاوره انجام آزمايشات، طراحي پرسشنامه، مديريت عمليات ميداني، مديريت داده‌ها، ورود اطلاعات، انجام آزمايشات و ...**

**3- محتوای علمی پژوهش**

**3-1- مساله پژوهش** (شامل اهميت، ضرورت و پرسش پژوهش)

سندروم روده تحریک پذیر یک اختلال عملکردی روده می باشد([1](#_ENREF_1))که با تغییر عادات دفع، تحریک پذیری بیش از حد نسبت به انبساط رکتوم و نفوذپذیری بیش از حد روده مشخص می شود ([2](#_ENREF_2), [3](#_ENREF_3)). متاسفانه هنوز درمان قطعی برای این اختلال شایع وجود ندارد ([4](#_ENREF_4)). پاتوژنز سندروم روده تحریک پذیر هنوز نامشخص است که احتمالا در اثر التهاب، تغییر میکروفلور کولون و رشد بیش از حد باکتریها می باشد([5](#_ENREF_5)), همچنین التهاب خفیف مزمن موکوس روده و عدم فعالیت مناسب ماست سلها از عوامل بالقوه اتیولوژی این بیماری هستند ([4](#_ENREF_4)). علاوه براین، افزایش اینترلوکینهای پیش التهابی پلاسما همچون اینترلوکین-1، اینترلوکین-6 و TNF-alpha در بیماران مبتلا به سندروم روده تحریک پذیر مشاهده شده است ([6](#_ENREF_6)). سایر اختلالات همراه با این بیماری شامل سردرد، دردهای مزمن عضلانی، سندروم خستگی مزمن و افسردگی هستند، همچنین خطر استئوپروز و شکستگی در این بیماران بیشتر می باشد([7](#_ENREF_7)). تا به حال درمانهای روانشناسی، دارویی و غذایی بسیاری برای سندروم روده تحریک پذیر مطرح شده اند ولی هیچیک درمان قطعی موثر نبوده اند ([5](#_ENREF_5))

زنان در سن باروری نسبت به مردان بیشتر در معرض ابتلا به سندروم روده تحریک پذیر قرار دارند که احتمالا با فشارهای روانی مرتبط می باشد([8](#_ENREF_8), [9](#_ENREF_9)). همچنین حساسیت به درد در زنان بیشتر است ([10](#_ENREF_10)). مطالعات حیوانی حاکی ازتغییر در نفوذپذیری روده در پاسخ به میزان متغیر هورمونهای زنانه از جمله استروژن می باشند ([11](#_ENREF_11)). در سندروم روده تحریک پذیر افزایش نفوذپذیری روده از عوامل موثر در شدت علایم بالینی می باشد ([12](#_ENREF_12)). در زنان مبتلا به سندروم روده تحریک پذیر میزان سرین-پروتئاز مدفوعی افزایش می یابد که نشان دهنده افزایش میزان نفوذپذیری غشای روده و نیز تحریک پذیری آن می باشد ([13](#_ENREF_13)).

گیرنده های استروژنی در دستگاه گوارشی بیان می شوند که میزان گیرنده های نوع بتا بیشتر در موکوس کولون می باشد ([14](#_ENREF_14)). بر اساس یافته های مطالعات حیوانی و انسانی استروژن از طریق گیرنده های استروژنی نوع بتا موجب ایجاد سد طبیعی اپیتلیال کولون می شود([11](#_ENREF_11), [14](#_ENREF_14)) ایزوفلاونهای استخراج شده سویا (فاقد فیبر سویا) فیتواستروژنهایی هستند که از نظر ساختمانی مشابه 17-بتا استرادیول می باشند و تمایل بالایی برای اتصال به گیرنده های استروژنی نوع بتای کولون دارند ([15](#_ENREF_15)). این ایزوفلاونها شامل دایدزئین، جنستئین و گلایستین هستند که به شکل دست نخورده قابل جذب از اپیتلیوم روده می باشند([16](#_ENREF_16)). علاوه بر این، ایزوفلاونهای سویا دارای یک مهارکننده پایدار پروتئاز به نام مهارگر بیرک بومن (Bowman Birk Inhibitor) یا (BBI) هستند که قادر به کاهش پروتئاز در روده و کاهش نفوذ پذیری بیش از حد روده می باشد([17](#_ENREF_17)). سویا می تواند با کاهش بیان ژن اینترلوکینهای پیش التهابی مانند اینترلوکین-1، 6 و12 بتا موجب کاهش التهاب کولون شود ([18](#_ENREF_18)).

ویتامین D_3_ یا کوله کلسیفرول می تواند به علت اثرات ضد تکثیری، محرک تمایز سلولی و تنظیم سیستم ایمنی و ضد التهابی در کنترل علایم بالینی سندروم روده تحریک پذیر موثر باشد ([5](#_ENREF_5)). علاوه بر تاثیر ویتامین D بر متابولیسم استخوان و مواد معدنی با اتصال به گیرنده ویتامین D در روده موجب مهار تکثیر مونونوکلئارها و لنفوسیتهای T و در نتیجه کاهش اینترلوکینهای پیش التهابی همچون اینترلوکین-1 بتا، 2 و 6 و نیز اینترفرون گاما و فاکتور نکروز تومور آلفا می شود ([5](#_ENREF_5)). همچنین کمبود ویتامین D در بیماران مبتلا به سندروم روده تحریک پذیر دیده شده است که احتمالا با مکملیاری با ویتامین D بتوان علایم بالینی آن را کاهش داد ([19](#_ENREF_19)).

ویتامین D نیز همانند سویا با اتصال به گیرنده های استروژن موجب تنظیم عملکرد آنها می شود به طوری که ویتامین D می تواند به گیرنده استروژن غشایی از نوع بتا و نیز گیرنده استروژن هسته ای از نوع آلفا متصل شود و موجب تنظیم عملکرد استروژنها در عضلات صاف روده گردد ([20](#_ENREF_20)). بنابراین به نظر می رسد که تجویز توام مکمل ایزوفلاونهای سویا همراه با ویتامین D_3_ اثر سینرژیستیک در تنظیم عملکرد گیرنده های استروژنی و نیز میزان نفوذپذیری غشای روده و کنترل علایم بالینی سندروم روده تحریک پذیر داشته باشد.

بر اساس منابع علمی در دسترس، تاکنون مطالعه کارآزمایی بالینی درباره تاثیر ایزوفلاونهای سویا و ویتامین D_3_ در بیماران مبتلا به سندروم روده تحریک پذیر انجام نشده است. هدف از این مطالعه تعیین تاثیرات تجویز مکمل کوله کلسیفرول و ایزوفلاونهای سویا به تنهایی و توام در مقایسه با دارونما بر روی سیتوکین التهابی TNF-alpha، شاخص تام آنتی اکسیدانی، علایم بالینی و کیفیت زندگی می باشد.

**3-2- هدف های پژوهش**

**هدف کلی :** تعیین تاثیر کوله کلسیفرول، ایزوفلاونهای سویا و تجویز توام انها در مقایسه با دارونما بر علایم بالینی، کیفیت زندگی، سیتوکین التهابی TNF-alpha، ظرفیت تام آنتی اکسیدانی سرم TAC و میزان نفوذپذیری روده در بیماران مبتلا به سندروم روده تحریک پذیر

**اهداف اختصاصی**

الف – تعیین متغیرهای وزن، BMI ، میزان کل انرژی، کربوهیدرات، فیبر، کل چربی، اسیدهای چرب اشباع، اسیدهای چرب غیر اشباع MUFA و PUFA امگا-3 و PUFA امگا-6، کلسترول رژیم غذایی، TNF-α و TAC ، علایم بالینی، کیفیت زندگی در شروع و پایان مطالعه در بیماران 4 گروه دریافت کننده مکمل ایزوفلاون سویا، ویتامین D ، ویتامین D- سویا توام و گروه دارونما.

ب – مقایسه میانگین غلظت سرمی TNF-α و TAC قبل و پس از مداخله در داخل گروهها و تغییرات میانگین آنها بین گروهها ج- مقایسه علایم بالینی، کیفیت زندگی بین و داخل گروهها قبل و بعد از مداخله

د- مقایسه میانگین غلظت ادراری لاکتولوز و مانیتول و نسبت آنها بین و داخل گروهها قبل و بعد از مداخله و تغییرات میانگین آنها بین گروهها

**هدف کاربردی**

ایجاد روش های درمانی موثر و ایمن جهت بهبود علایم بالینی، کیفیت زندگی، سیتوکین التهابی TNF-alpha، شاخص ظرفیت تام آنتی اکسیدانی سرمی و میزان نفوذپذیری روده در بیماران مبتلا به سندروم روده تحریک پذیر

**هدف نهایی**

ارتقای سطح سلامت بیماران مبتلا به سندروم روده تحریک پذیر

**3-3- فرضيه‌های پژوهش**

الف – مصرف توام مکمل ایزوفلاون سویا و ویتامین D در مقایسه با دارونما و نیز هریک از مکملها به تنهایی باعث کاهش غلظت سیتوکین التهابی TNF-α در PBMC می گردد.

ب - مصرف توام مکمل ایزوفلاون سویا و ویتامین D در مقایسه با دارونما و نیز هریک از مکملها به تنهایی باعث افزایش غلظت TAC سرم می گردد.

ج- مصرف توام مکمل ایزوفلاون سویا و ویتامین D در مقایسه با دارونما و نیز هریک از مکملها به تنهایی باعث بهبود علایم بالینی و کیفیت زندگی می شود.

د- مصرف توام مکمل ایزوفلاون سویا و ویتامین D در مقایسه با دارونما و نیز هریک از مکملها به تنهایی باعث کاهش میزان مانیتول، لاکتولوز ادراری و کاهش نسبت لاکتولوز به مانیتول ادراری و در نتیجه کاهش نفوذپذیری روده کوچک می شود.

**3-4- جنبه(هاي) نوآوري و دستاورد(هاي) ويژه طرح** (در صورتي‌كه طرح داراي دستاوردها و نوآوري‌هاي ملي و يا بين‌المللي است يا به دانش فني و فن‌آوري قابل اجرا در كشور منتهي مي‌شود، به طور خاص، تشريح فرماييد)

بر اساس بررسی منابع علمی موجود، تاکنون مطالعه کارآزمایی بالینی مبنی بر بررسی تاثیر توام مکمل ایزوفلاون سویا و ویتامین D در بیماران مبتلا به سندروم روده تحریک پذیر وجود ندارد. در صورت تایید اثر مثبت مصرف توام مکمل ایزوفلاون سویا و ویتامین D در مقایسه با دارونما می توان تولید نوشیدنی بر پایه سویا غنی شده با ویتامین D را به منظور مصرف معمول در بیماران زن مبتلا به سندروم روده تحریک پذیر پیشنهاد کرد.

**3-5- تعريف‌ها و مفهوم‌هاي علمي ضروري در ارتباط با موضوع پژوهش (در صورت نياز)**

**3-6- سابقه تحقيقاتي عنوان و موضوع مورد پژوهش**

بر اساس منابع علمی موجود، هیچ مطالعه انسانی بر روی تاثیر ایزوفلاونهای سویا و یا ویتامین D در بیماران مبتلا به سندروم روده تحریک پذیر انجام نشده است و تنها چند مطالعه اندک درباره تاثیر مثبت آنها بر نفوذپذیری غشای روده در رتهای مونث مبتلا به سندروم روده تحریک پذیر وجود دارد.

مطالعه Moussa و همکاران با سویای جوانه زده تخمیر شده به مدت 2 هفته بر روی رتهای مونث مبتلا به سندروم روده تحریک پذیر انجام دادند، نشان داد که ایزوفلاونهای سویا به میزان 45/0 میلی گرم به ازای روز (55٪ دایدزئین، 30٪ گلایستین، 15٪ جنستئین ) در مقایسه با گروه کنترل، با فعال سازی گیرنده استروژنی روده و کاهش میزان فعالیت پروتئاز مدفوع موجب کاهش نفوذپذیری بیش از حد غشای روده تحت استرس و کاهش حساسیت بیش از حد احشایی با افزایش بیان ژن اکلودین و کاهش میزان ماست سلهای کولون می شود ([21](#_ENREF_21)).

مطالعه دیگری که همین محققین(Moussa و همکاران) با سویای جوانه زده تخمیر شده به همان میزان فوق (55٪ دایدزئین، 30٪ گلایستین، 15٪ جنستئین ) در مقایسه با گروه کنترل، به مدت 2 هفته بر روی رتهای مونث مبتلا به بیماری التهابی روده انجام دادند نشان داد که ایزوفلاونهای سویا یا تحریک مسیر گیرنده استروژنی و افزایش بیان مهار کننده گیرنده سرین-پروتئاز-2 در ایلئوم وکولون موجب کاهش اینترلوکینهای پیش التهابی و در نتیجه کاهش علایم ماکروسکوپیک و میکروسکوپیک هیستولوژیک التهاب روده می شود ([22](#_ENREF_22)).

مطالعه Sprake و همکاران که یک گزارش موردی از خانم 41 ساله مبتلا به 25 سال سندروم روده تحریک پذیر با بروز غالب اسهال بود بیان گر این مطلب است که علی رغم تست بسیاری از مکملها و روشهای طب کمکی هنوز علایم بالینی فرد مبتلا بهبود نیافته بود ولی با مصرف منظم 1000 واحد در فصل تابستان و 3000 تا 4000 واحد ویتامین D در فصل زمستان علایم بالینی او سرکوب شده بود با وجود تاثیر بالقوه ویتامین D در درمان سندروم تحریک پذیر روده نمی تواند روش درمانی قابل توصیه در همه انواع زیر گروههای این بیماری باشد که نیاز به در نظر گرفتن شاخصه های ژنتیکی متفاوت بین افراد مبتلا می باشد همچنین بر اساس ژنتیک افراد می توان میزان پاسخ دهی به مکملیاری با ویتامین D را پیش بینی کرد و احتمال مسمومیت با مکمل در برخی بیماران فاقد کمبود ویتامین D دور از ذهن نحواهد بود، البته به منظور ارزیابی اثربخشی مکمل ویتامین D در بهبود سندروم روده تحریک پذیر نیاز به انجام کارآزمایی بالینی وجود دارد ([19](#_ENREF_19))

از طرف دیگر بنا به نظر Zender و همکاران احتمال ابتلا به استرس و اضطراب در جنس مونث نسبت به مذکر بالاتر است بیماریهای همراه با افسردگی و استرس در زنان شامل سندروم روده تحریک پذیر، کمبود ویتامین D، بیماریهای قلبی و استرس بیمارگونه است ([23](#_ENREF_23))

همچنین مطالعه Young و همکاران با پپتیدهای سویا به مدت 5 روز بر روی بچه خوکهای مبتلا به کولیت حاکی از تاثیر معنی دار سویا بر کاهش اینترلوکینهای پیش التهابی اینترلوکین-1 بتا، TNF و اینترلوکین-17 آلفا و در نهایت کاهش میزان التهاب کولون بود ([18](#_ENREF_18)).

این نتایج مقدماتی دلالت کننده محکمی بر توانایی بالای ایزوفلاونهای سویا و ویتامین D در کنترل سندروم روده تحریک پذیر دارد. همچنین با مقایسه تاثیر ایزوفلاونهای سویا در دو مطالعه Moussa و همکاران، به این نتیجه رسیدیم که با وجود عدم تغییر میزان کورتیکواسترون پلاسما و تاثیر مثبت در گروه مداخله، احتمالا ایزوفلاونهای سویای تجویزی با اتصال به لیگاندهای ER موجب کاهش حساسیت بیش از حد عروق روده ای تحت تاثیر استرس روانی می شوند علاوه براین، به علت عدم امکان پرکردن پرسشنامه در مطالعات تجربی و عدم جمع آوری داده های نظرسنجی و کیفی تعیین تاثیر مکملیاری در بیماران مبتلا به سندروم روده تحریک پذیر با وجود عوامل مخدوشگر متعدد نیاز به مطالعات با متدولوژی صحیح و قابل تعمیم دارد و از آنجایی که مطالعه کارآزمایی بالینی در این زمینه فعلا صورت نگرفته است و مطالعه مشابه حیوانی در سال 2013 چاپ شده است نیاز به انجام این مطالعه انسانی ضروری به نظر می رسد و نیاز به مطالعات بالینی برای شناسایی بهتر مکانیسمها و کاربرد بالینی آنها مورد نیاز است. از سوی دیگر هزینه کم ، ایمن بودن و پذیرش مناسب این مکملها توسط اکثر بیماران نشانگر مناسب بودن این شیوه درمانی در افراد مبتلا می باشد.

**4- طرح مطالعاتی(روش و نحوه اجراي تحقيق)**

**4-1- متغيرهاي تحقيق**

متغيرهاي اصلي و مهم را در جدول زير وارد كنيد.

| متغير | ^*^نقش متغير | ^**^نوع متغير | مقیاس سنجش متغیر | اساس روش آزمون  /ابزار سنجش | مرجع | مکان انجام آزمایش |
| --- | --- | --- | --- | --- | --- | --- |
| نوع مکمل دریافتی | مستقل | کیفی اسمی | 1. ایزوفلاون سویا با ویتامین D 2. ایزوفلاون سویا 3. ویتامین D   دارونما | فرم جمع آوری داده ها |  | کلینیک بيماريهاي گوارش و کبد دانشگاه تهران |
| زمان اندازه گیری | مستقل | کیفی اسمی | 1. قبل از مداخله 2. بعد از مداخله | فرم جمع آوری داده ها |  | کلینیک بيماريهاي گوارش و کبد دانشگاه تهران |
| میزان مواجهه با نور خورشید | مخدوش کننده | کمی پیوسته | دقیقه | پرسشنامه |  | کلینیک بيماريهاي گوارش و کبد دانشگاه تهران |
| سن | مخدوش کننده | کمی گسسته | سال | پرسش |  | کلینیک بيماريهاي گوارش و کبد دانشگاه تهران |
| قد | مخدوش کننده | کمی پیوسته | سانتی متر | متر نواری نصب شده بر روی دیوار |  | کلینیک بيماريهاي گوارش و کبد دانشگاه تهران |
| وزن | مخدوش کننده | کمی پیوسته | کیلوگرم | ترازو |  | کلینیک بيماريهاي گوارش و کبد دانشگاه تهران |
| نمایه توده بدن  (BMI) | مخدوش کننده | کمی پیوسته | نسبت وزن (بر حسب کیلوگرم) به توان دوم قد (بر حسب متر) | محاسبه |  | کلینیک بيماريهاي گوارش و کبد دانشگاه تهران |
| سیگاری بودن | مخدوش کننده | کیفی اسمی | 1. سیگاری   غیر سیگاری | پرسش |  | کلینیک بيماريهاي گوارش و کبد دانشگاه تهران |
| یائسگی | مخدوش کننده | کیفی اسمی | 1. یائسه 2. غیر یائسه | پرسش |  | کلینیک بيماريهاي گوارش و کبد دانشگاه تهران |
| کل انرژی دریافتی | مخدوش کننده | کمی پیوسته | Kcal/d | پرسشنامه یادآمد خوراک |  | کلینیک بيماريهاي گوارش و کبد دانشگاه تهران |
| کربوهیدرات دریافتی | مخدوش کننده | کمی پیوسته | g/d | پرسشنامه یادآمد خوراک |  | کلینیک بيماريهاي گوارش و کبد دانشگاه تهران |
| کلسترول دریافتی | مخدوش کننده | کمی پیوسته | Mg/d | پرسشنامه یادآمد خوراک |  | کلینیک بيماريهاي گوارش و کبد دانشگاه تهران |
| کل چربی دریافتی | مخدوش کننده | کمی پیوسته | g/d | پرسشنامه یادآمد خوراک |  | کلینیک بيماريهاي گوارش و کبد دانشگاه تهران |
| فیبر دریافتی | مخدوش کننده | کمی پیوسته | g/d | پرسشنامه یادآمد خوراک |  | کلینیک بيماريهاي گوارش و کبد دانشگاه تهران |
| اسیدهای چرب SFA دریافتی | مخدوش کننده | کمی پیوسته | g/d | پرسشنامه یادآمد خوراک |  | کلینیک بيماريهاي گوارش و کبد دانشگاه تهران |
| اسیدهای چرب MUFA دریافتی | مخدوش کننده | کمی پیوسته | g/d | پرسشنامه یادآمد خوراک |  | کلینیک بيماريهاي گوارش و کبد دانشگاه تهران |
| اسیدهای چرب PUFA- امگا 3 دریافتی | مخدوش کننده | کمی پیوسته | g/d | پرسشنامه یادآمد خوراک |  | کلینیک بيماريهاي گوارش و کبد دانشگاه تهران |
| اسیدهای چرب PUFA- امگا 6 دریافتی | مخدوش کننده | کمی پیوسته | g/d | پرسشنامه یادآمد خوراک |  | کلینیک بيماريهاي گوارش و کبد دانشگاه تهران |
| ویتامین E دریافتی | مخدوش کننده | کمی پیوسته | Mg/d | پرسشنامه یادآمد خوراک |  | کلینیک بيماريهاي گوارش و کبد دانشگاه تهران |
| ویتامین C دریافتی | مخدوش کننده | کمی پیوسته | Mg/d | پرسشنامه یادآمد خوراک |  | کلینیک بيماريهاي گوارش و کبد دانشگاه تهران |
| روی (Zn) دریافتی | مخدوش کننده | کمی پیوسته | Mg/d | پرسشنامه یادآمد خوراک |  | کلینیک بيماريهاي گوارش و کبد دانشگاه تهران |
| سلنیم (Se) دریافتی | مخدوش کننده | کمی پیوسته | Mg/d | پرسشنامه یادآمد خوراک |  | کلینیک بيماريهاي گوارش و کبد دانشگاه تهران |
| کلسیم دریافتی | مخدوش کننده | کمی پیوسته | Mg/d | پرسشنامه یادآمد خوراک |  | کلینیک بيماريهاي گوارش و کبد دانشگاه تهران |
| ویتامین D دریافتی | مخدوش کننده | کمی پیوسته | IU/d | پرسشنامه یادآمد خوراک |  | کلینیک بيماريهاي گوارش و کبد دانشگاه تهران |
| TNF-α | وابسته | کمی پیوسته | Pg/ml | کیت الایزا |  | کلینیک بيماريهاي گوارش و کبد دانشگاه تهران |
| TAC | وابسته | کمی پیوسته | mMol | کیت الایزا |  | کلینیک بيماريهاي گوارش و کبد دانشگاه تهران |
| میزان مانیتول ادراری | وابسته | کمی پیوسته | میلی گرم بر دسی لیتر | HPLC و ااسپکترومتری |  | کلینیک بيماريهاي گوارش و کبد دانشگاه تهران |
| میزان لاکتولوز ادراری | وابسته | کمی پیوسته | میلی گرم بر دسی لیتر | HPLC و ااسپکترومتری |  | کلینیک بيماريهاي گوارش و کبد دانشگاه تهران |
| نسبت لاکتولوز به مانیتول ادراری | وابسته | کمی پیوسته | بدون واحد | محاسبه |  | کلینیک بيماريهاي گوارش و کبد دانشگاه تهران |
| 25-هیدروکسی ویتامین D سرمی | وابسته | کمی پیوسته | ng/ml | کیت الایزا |  | کلینیک بيماريهاي گوارش و کبد دانشگاه تهران |

**4-2- جداول توخالي اصلي مربوط به گزارش داده‌هاي طرح** (جداول مربوط، در پيوست طرح- بند 9- ارائه شود)

**4-3- پرسشنامه ها يا فرم‌هاي اطلاعاتي** (جداول مربوط، در پيوست طرح- بند 9- ارائه شود)

**4-4- مواد و روش ها**

**4-4-1- مواد مصرفی**

| **نوع ماده مصرفي** | **شركت سازنده (شناسه ماده/شركت/كشور)** |
| --- | --- |
|  |  |
|  |  |

**4-4-2- مواد غیر مصرفی**

| **نام دستگاه/ابزار** | **مدل/شركت/كشور** |
| --- | --- |
|  |  |
|  |  |

**4-5- جامعه و نمونه مورد بررسي**

**شامل (حجم نمونه و روش نمونه‌گيري) يا شرح تهيه نمونه‌ها**

# نوع مطالعه

این تحقیق یک کارآزمایی بالینی تصادفی دو سو کور (Randomized Clinical Trial Double Blind) می باشد.

# جمعیت هدف

بیماران مبتلا به سندروم روده تحریک پذیر

# نمونه مورد مطالعه

بیماران مبتلا به سندروم روده تحریک پذیر مراجعه کننده به کلنیک های مشاوره و درمان بیماری های کبد و دستگاه گوارش که مطابق با معیارهای زیر انتخاب می شوند

**الف – معیارهای ورود به مطالعه**

- سن 18 تا 65 سال
- بیماران مبتلا به سندروم روده تحریک پذیر بنا به تشخیص متخصص گوارش طبق معیارهای ROME-III (1- آسودگی بعد از دفع مدفوع، 2- تغییر در تکرر دفع مدفوع، 3- تغییر در شکل و قوام مدفوع، حداقل در سه روز از ماه، سه ماه در سال و عدم وجود یافته پاتولوژیک در بررسی های گوارشی)
- نمایه توده بدنی در دامنه نرمال (25-18)
- عدم هرگونه بیماری ارگانیک روده ای (تشخیص بر اساس کولونوسکوپی در 5 سال گذشته) و عفونت روده ای (تشخیص بر اساس کشت مدفوع در نمونه مشکوک)
- عدم تاریخچه پزشکی بیماری مزمن گوارشی و کولورکتال
- عدم هرگونه جراحی عمده روده
- عدم مصرف منظم داروهای مسهل یا ضد اسهال
- عدم مصرف مزمن آنتی بیوتیکها و کورتیکواستروییدها و سرکوب کننده های ایمنی
- عدم مصرف داروهای تغییردهنده حرکت گوارشی مانند متوکلوپرامید، سیزاپراید، مواد مخدر، دیفنوکسیلات و...
- عدم مصرف داروهای افزاینده خونریزی از موکوس روده مانند آسپرین، وارفارین، هپارین، ...
- عدم بارداری یا شیردهی، ورزشكار یا بستري بودن
- عدم سابقه ابتلا به سرطان پستان در خود فرد یا بستگان درجه یک خانواده
- عدم اختلال شدید روانی ورفتاری
- عدم مصرف نیکوتین و مشتقات آن در 6 ماه گذشته
- عدم مصرف داروهای ضدالتهابی غیر استروییدی و آسپرین در هفته گذشته (اثر بر نفوذپذیری روده)
- **ب – معیارهای خروج از مطالعه**
- مصرف مکمل ایزوفلاون سویا یا ویتامین D از یک سال قبل از ورود به مطالعه و حین آن
- مصرف فرآورده های حاوی سویا مانند شیر سویا و آجیل سویا در طول مطالعه
- تغییر رژیم غذایی در طول مطالعه
- مصرف شیرین کننده های مصنوعی(اثر بر نفوذپذیری روده) در 2 روز قبل از ورود به مطالعه و حین آن
- عدم ادامه مطالعه به دلیل عارضه جانبی دارو
- عدم تمایل به ادامه همکاری
- بارداری حین مطالعه

- **روش نمونه گیری و حجم نمونه**
- برای محاسبه حجم نمونه برای این مطالعه که از نوع فاکتوریال می باشد بایستی ابتدا حجم نمونه موردنیاز برای مقایسه ی گروه دریافت کننده سویا در مقابل گروه کنترل و نیز گروه دریافت کننده ی ویتامین D در مقابل گروه کنترل محاسبه شده و ماکزیمم این دو به عنوان حجم نمونه نهایی درنظر گرفته شود که این مقدار حجم نمونه برای ارزیابی اثرات اصلی کفایت می کند. براساس اطلاعات بدست آمده از مطالعه (24) که در زیر ارایه شده است، تعداد حجم نمونه لازم برای مقایسه گروه دریافت کننده سویا در مقابل گروه کنترل براساس آزمون تی برای مقایسه ی میانگین های کیفیت زندگی و احتمال خطای نوع اول 5درصد و احتمال خطای نوع دوم 20 درصد و نیز اطلاعات ارایه شده مقاله (24) برابر 21 نفر در هر گروه بدست آمد که برای دقت بیشتر، محاسبات با استفاده از نرم افزار GPower نسخه ی 3.1.7 صورت گرفت که خروجی آن پیوست می باشد. هیچ مطالعه ای در مورد اثر مکمل ویتامین D در برابر گروه کنترل در این بیماران یافت نشد و لذا با فرض اثر مشابه مکمل سویا برای این گروه همان تعداد 21 نفر در هر گروه درنظر گرفته شد. با در نظر گرفتن احتمال ریزش برابر 15 درصد تعداد نمونه به 25 نفر در هر گروه و مجموع 100 نفر افزایش یافت. لازم به ذکر است که این میزان حجم نمونه برای یافتن اثر متقابلی معادل 2 برابر اثرات اصلی کفایت می کند و برای یافتن اثرات متقابل کوچکتر نیاز به حجم نمونه های بالاتری است ولی با توجه به محدودیت های هزینه و اجرا امکان افزایش تعداد نمونه وجود ندارد. همسان سازی بیماران از نظر سن و یائسگی و نوع داروهای مصرفی انجام خواهد گرفت.
- n_1_ = 10 S_1_ = 81/1
- n_2_ = 9 S_2_ = 77/1
- µ_1_ - µ_2_ = 60/1
- α= 05/0
- β= 20/0

$$N=\left[2(SD)^{2}(Z_{\frac{\alpha}{2}}+Z_{\beta})^{2}\right]/\Delta^{2}$$

در این مطالعه نمونه­ها از کلنیک های مشاوره و درمان بیماری های کبد و دستگاه گوارش با روش نمونه­گیری آسان (Convenience Sampling) انتخاب می­گردند. به منظور تصادفی سازی ورود نمونه ها به هر یک از گروهها، بر روی پاکتهای حاوی مکمل و دارونما نامگذاری به صورت A، B، C و D انجام خواهد گرفت و قبل از شروع مطالعه، مجموعه قوطی­های حاوی کپسول­های مربوطه، توسط فردی غیر از پژوهشگر بصورت گروه A (AD, AS) حاوی مکمل سویا و دارونمای ویتامین D، گروه B (BD, BS) حاوی مکمل سویا و مکمل ویتامین D، گروه C (CD, CS)حاوی دارونمای سویا و دارونمای ویتامین D، گروه (DD, DS) D حاوی مکمل ویتامین D و دارونمای سویا کدگذاری می­شوند تا عدم اطلاع محقق از نوع کپسول­های دریافتی، توسط هر گروه، مراعات گردد.

**4-6- روش تحقیق، شرح عملياتي و تفصيلي انجام كار**

در این مطالعه از بیماران مبتلا به سندروم روده تحریک پذیر مراجعه کننده به کلنیک های مشاوره و درمان بیماری های دستگاه گوارش که دارای معیارهای ورود به مطالعه هستند، در صورت تمایل برای شرکت در این تحقیق ، پس از 12 تا 14 ساعت ناشتایی ، 5 سی سی خون گرفته می شود و سرم خون آنها جهت اندازه گیری غلظت فاکتور التهابی و سایر فراسنج های بیوشیمیایی سرم در فریزر نگهداری می شوند. در شروع مطالعه ، ابتدا اهداف و روش اجرای مطالعه برای بیماران توضیح داده می شود و سپس از کلیه بیماران داوطلب رضایت نامه کتبی گرفته می شود (پیوست 1). شرکت کنندگان به روش بلوکه بندی شده تصادفی متعادل شده به 4 گروه دریافت کننده مکمل ایزوفلاون سویا، ویتامین D ، ایزوفلاون سویا-ویتامین D یا گروه دارونما تقسیم می شوند. همچنین در شروع مطالعه وزن، قد هر بیمار اندازه گیری می شوند و BMI با استفاده از فرمول (تقسیم وزن به کیلوگرم بر مجذور قد به مترمربع) بدست خواهد آمد. برگه مشخصات عمومی برای هر بیمار تکمیل می گردد (پیوست 2) و نیز پرسشنامه معتبر علایم بالینی و کیفیت زندگی در ابتدای مطالعه و پایان هفته ششم تکمیل می شود. در این مطالعه به منظور بررسی رژیم غذایی بیماران، در ابتدای مطالعه و پایان هفته ششم از بیماران سه روز یادآمد خوراک 24 ساعته در مورد یک روز تعطیل و دو روز غیر تعطیل، از طریق مصاحبه حضوری و تلفنی تکمیل می­شود (پیوست3). تجزیه و تحلیل پرسشنامه­های یادآمد خوراک 24 ساعته، با استفاده از نرم افزار تغذیه­ای Nutritionist IV (N4) صورت خواهد گرفت.

در این مطالعه بیماران برحسب گروهی که در آن قرار می گیرند به مدت 6 هفته مکمل های مربوطه را دریافت می نمایند. بیماران در گروه دریافت کننده مکمل ایزوفلاون سویا ساخت شرکت 21th Century Co. ساخت کشور ایالات متحده ، روزانه دو کپسول حاوی 20 میلی گرم ایزوفلاون ([24](#_ENREF_24), [25](#_ENREF_25))که شامل 10 میلی گرم دایدزئین، 5/8 میلی گرم جنستئین و 5/1 میلی گرم گلایستین می باشد همراه با دارونمای دوهفته ای یکبار ویتامین D ساخت شرکت زهراوی ایران دریافت خواهند کرد، همچنین گروه دریافت کننده مکمل ویتامین D به میزان 50000 واحد در دو هفته ([26](#_ENREF_26)) همراه با دارونمای ایزوفلاون سویا دریافت خواهند کرد و گروه دریافت کننده مکمل ایزوفلاون سویا توام با ویتامین D مکمل روزانه ایزوفلاون سویا با مکمل دوهفته ای ویتامین D دریافت خواهند کرد در حالی که به بیماران گروه دارونما روزانه دو کپسول دارونمای ایزوفلاون سویا و یک دارونمای دوهفته ای ویتامین D داده می شود که این کپسول ها از نظر ظاهری، مشابه کپسول مکمل همنام خود می­باشد. کپسول­های مکمل و دارونما توسط شرکتهای سازنده تهیه می­گردند و از نشاسته در کپسول­های دارونمای ایزوفلاون سویا و از MCT oil در کپسولهای دارونمای ویتامین D استفاده می­شود. قبل از شروع مطالعه، مجموعه قوطی­های حاوی کپسول­های مربوطه، توسط فردی غیر از پژوهشگر بصورت گروه A (AD, AS)، گروه B (BD, BS) ، گروه C (CD, CS)، گروه (DD, DS) D کدگذاری می­شوند تا عدم اطلاع محقق از نوع کپسول­های دریافتی، توسط هر گروه، مراعات گردد. قوطی­های حاوی کپسول­ در شروع مطالعه به تعداد کافی به بیماران داده می­شود و از آن­ها خواسته می­شود هر روز دو عدد کپسول مکمل یا دارونمای ایزوفلاون سویا و نیز دوهفته ای یکبارکپسول مکمل با دارونمای ویتامین D مصرف نمایند. در پایان مطالعه با اندازه گیری مجدد وزن بیماران ، BMI آن­ها محاسبه اندازه گیری می­شود و از بیماران پس از 12 تا 14 ساعت ناشتایی 5 سی سی نمونه خون گرفته می­شود. نمونه های خون گرفته شده از بیماران در شروع مطالعه و پایان هفته ششم به مدت 10 دقیقه با سرعت 2000-1500 دور در دقیقه (معادل 26/1 تا 24/2 G یا RCF) سانتریفوژ می شوند (طبق فرمول **RCF or G-force= 1.12  x  R  x  (RPM/1000)²**) تا سرم آنها جدا گردد. همچنین PBMC از خون تازه بیماران جداسازی شده و تا روز انجام آزمایشها در فریزر C◦80 – نگهداری می شود و غلظت TNF-α در PBMC به روش الایزا و غلظت سرمی TAC به روش الایزا با استفاده از کیت مربوطه ، میزان نفوذپذیری روده به روش میزان دفع ادراری مانیتول و لاکتولوز (بعد از خوردن 5 گرم بر دسی لیتر لاکتولوز و 1 گرم بر دسی لیتر مانیتول با رعایت ناشتا بودن تا 5 ساعت و دریافت بدون محدودیت آب در یک ظرف دربسته جمع آوری و نگهداری در فریزر C◦80 – تا روز اندازه گیری) به روش HPLC و اسپکترومتری جرمی اندازه گيري مي شود. همچنین، سطح سرمی 25-هیدروکسی ویتامین D با کمک کیت الایزا در ابتدای مطالعه و پایان هفته ششم به منظور تعیین وضعیت پایه ویتامین D و نیز ارزیابی میزان مصرف کپسولها اندازه گیری می شود و از پرسشنامه تعیین دریافت ویتامین D از نورخورشید نیز استفاده می شود.

پیگیری بیماران در این پژوهش، به منظور کنترل آن­ها از نظر مصرف کپسول­ها و جلوگیری از ریزش نمونه­ها تقریبا هر هفته یکبار بصورت تلفنی انجام خواهد شد و در پایان هفته ششم نیز با شمارش کپسول­های باقیمانده میزان رعایت بیماران از نظر مصرف کپسول­ها مورد ارزشیابی قرار می­گیرد و بیمارانی که بیش از 10 درصد کپسول­های خود را مصرف نکرده باشند از تحقیق کنار گذاشته می­شوند. همچنین تغییر عمده در رژیم غذایی یا فعالیت فیزیکی در طول مطالعه موجب حذف از مطالعه می شود.

**4-7- طرح آماري پژوهش**

**(روش آماري پژوهش، گردآوري داده‌ها و ارزيابي آماري)**

در این مطالعه، تجزیه و تحلیل آماری داده­ها از طریق نرم­افزار SPSS نسخه 17 صورت می­گیرد. برای توصیف داده های کمی از گزارش میانگین ( انحراف معیار) و در صورت نیاز از میانه (دامنه میان چارکی ) و برای توصیف داده های کیفی از گزارش فراوانی ( درصد) استفاده خواهد شد. جهت مقایسه متغیرهای کیفی مخدوش­کننده مانند جنس بین دو گروه از آزمون Chi Square و برای مقایسه توزیع متغیرهای کمی در گروهها از آنالیز واریانس یک طرفه استفاده می­گردد. جهت مقایسه میانگین متغیرهای کمی مخدوش­کننده تن­سنجی، زمینه ای، نورآفتاب و رژیمی در هر گروه از آزمون paired t test استفاده می­گردد و برای مقایسه میانگین آن­ها بین 4 گروه از آزمون ANOVA استفاده می شود. و در صورتیکه توزیع آن­ها نرمال نباشد، جهت مقایسه آن­ها در هر گروه از آزمون Wilcoxon و برای مقایسه آن­ها بین چهار گروه از آزمون Kruskal-Wallis استفاده می­گردد. جهت از بین بردن اثرات فاکتورهای مخدوش­کننده نیز از آزمون آنالیز کوواریانس و مدل رگرسیون استفاده خواهد شد. سطح معنی داری آماری در تمامی آنالیزها به صورت <0.05 p تعریف می گردد.

**5- جدول زمان بندي برنامه تحقيق**

شامل مهمترين مراحل طرح و تعيين زمان ارائه گزارش‌هاي پيشرفت

| رديف | شرح هر يك از  فعاليت‌هاي اجرايي طرح به تفكيك يا ارائه گزارش پيشرفت | **طول مدت (ماه)** | 24 ماه | | | | | | | | | | | | | | | | | | | | | | | |
| --- | --- | --- | --- | --- | --- | --- | --- | --- | --- | --- | --- | --- | --- | --- | --- | --- | --- | --- | --- | --- | --- | --- | --- | --- | --- | --- |
|  |  |  | 1 | 2 | 3 | 4 | 5 | 6 | 7 | 8 | 9 | 10 | 11 | 12 | 13 | 14 | 15 | 16 | 17 | 18 | 19 | 20 | 21 | 22 | 23 | 24 |
|  | تهیه کیت­ها و امکانات آزمایشگاهی جهت تعیین متغیرهای بیوشیمیایی سرم | 3 |  |  |  |  |  |  |  |  |  |  |  |  |  |  |  |  |  |  |  |  |  |  |  |  |
|  | گزارش پیشرفت 1 |  |  |  |  |  |  |  |  |  |  |  |  |  |  |  |  |  |  |  |  |  |  |  |  |  |
|  | هماهنگی با کلنیک بیماری های کبد و دستگاه گوارش ، انجام نمونه­گیری | 8 |  |  |  |  |  |  |  |  |  |  |  |  |  |  |  |  |  |  |  |  |  |  |  |  |
|  | گزارش پیشرفت 2 |  |  |  |  |  |  |  |  |  |  |  |  |  |  |  |  |  |  |  |  |  |  |  |  |  |
|  | اجرای برنامه مداخله | 8 |  |  |  |  |  |  |  |  |  |  |  |  |  |  |  |  |  |  |  |  |  |  |  |  |
|  | انجام آزمایشات بیوشیمیایی و مولکولی | 5/11 |  |  |  |  |  |  |  |  |  |  |  |  |  |  |  |  |  |  |  |  |  |  |  |  |
|  | گزارش پیشرفت 3 |  |  |  |  |  |  |  |  |  |  |  |  |  |  |  |  |  |  |  |  |  |  |  |  |  |
|  | تجزیه و تحلیل آماری داده­ها | 2 |  |  |  |  |  |  |  |  |  |  |  |  |  |  |  |  |  |  |  |  |  |  |  |  |
|  | تهیه گزارش نهایی | 3 |  |  |  |  |  |  |  |  |  |  |  |  |  |  |  |  |  |  |  |  |  |  |  |  |
|  | نظارت بر حسن اجرای  تحقیق (پایش یا Monitoring) | 24 |  |  |  |  |  |  |  |  |  |  |  |  |  |  |  |  |  |  |  |  |  |  |  |  |

**6- جدول بودجه بندي طرح**

**6-1- نيروي انساني**

| **رديف** | **نوع فعاليت** | **عنوان شغل** | **تعداد افراد** | **ساعات مورد نياز** | **حق الزحمه در ساعت (ريال)** | **جمع**  **(ريال)** |
| --- | --- | --- | --- | --- | --- | --- |
|  |  |  |  |  |  |  |
|  |  |  |  |  |  |  |
| **جمع** (ريال) | | | | | |  |

**6-2- هزينه آزمايشات و خدمات تخصصي كه توسط ديگر مؤسسات صورت مي گيرد:**

| **رديف** | **موضوع آزمايش يا خدمات تخصصي** | **مركز سرويس دهنده** | **تعداد كل دفعات** | **هزينه براي هر دفعه (ريال)** | **جمع**  **(ريال)** |
| --- | --- | --- | --- | --- | --- |
|  | خونگیری | آزمایشگاه تحصیلات تکمیلی دانشکده | 200 | 10000 | 2000000 |
|  | آماده سازی اولیه نمونه های خون و ادرار | آزمایشگاه تحصیلات تکمیلی دانشکده | 200 | 10000 | 2000000 |
|  | انجام HPLC | آزمایشگاه جامع تحقیقات داروسازی | 200 | 150000 | بدون هزینه30000000 |
|  | انجام اسپکترومتری جرمی | آزمایشگاه جامع تحقیقات داروسازی | 200 | 100000 | 20000000بدون هزینه |
|  | اندازه گیری 25 هیدروکسی ویتامین D سرمی | بیمه بیمار | 200 | - | - |
| **جمع** (ريال) | | | | | 4000000 |

**6-3- فهرست وسايل و مواد مصرفي كه بايد خريداري شود :**

| **ردیف** | **نام دستگاه و یا مواد** | **شرکت سازنده و یا فروشنده** | **کشور** | **مصرفی یا غیرمصرفی** | **آیا در ایران موجود است** | **تعداد لازم** | **قیمت واحد**  **(ریال)** | **قیمت کل**  **(ریال)** |
| --- | --- | --- | --- | --- | --- | --- | --- | --- |
| 1 | مکمل ایزوفلاون سویا | 21 century | امریکا | مصرفی | بله | 4500 | 10000 | 000/000/35 |
| 2 | مکمل ویتامین D | زهراوی | ایران | مصرفی | بله | 4500 | 1000 | 000/000/5 |
| 3 | کیت TNF-α | Cayman | آمریکا | مصرفی | بله | 2 | 000/000/15 | 000/000/30 |
| 4 | کیت TAC | Cayman | آمریکا | مصرفی | بله | 2 | 000/000/15 | 000/000/30 |
| 14 | ميكروتيوب 0/5 ml ,Dnase,Rnase free | طوبی نگین- ORANGE | دانمارک | مصرفی | بله | 1بسته | 000/400 | 000/400 |
| 15 | ميكروتيوب 0/2 ml ,Dnase,Rnase free | طوبی نگین- ORANGE | دانمارک | مصرفی | بله | 2بسته | 000/300 | 000/600 |
| 16 | سر سمپلر کریستالی | طوبی نگین- ORANGE | دانمارک | مصرفی | بله | 3بسته | 000/375 | 000/125/1 |
| 17 | لاکتولوز | Sigma | امریکا | مصرفی | بله | 2 بسته | 1000000 | 2000000 |
| 18 | مانیتول | Sigma | امریکا | مصرفی | بله | 2 بسته | 1000000 | 2000000 |
| 19 | دستکش یکبار مصرف بدون پودر | HMS | مالزی | مصرفی | بله | 2 بسته | 200000 | 400000 |
| جمع هزینه های وسایل و مواد (ریال) | | | | | | | | **10202500** |

**6-4- فهرست وسايل و مواد غيرمصرفي كه بايد خريداري شود :**

| **رديف** | **نام دستگاه يا مواد** | **شركت سازنده و يا فروشنده** | **كشور** | **آيا در ايران موجود است** | **تعداد لازم** | **قيمت واحد (ريال)** | **قيمت كل**  **(ريال)** |
| --- | --- | --- | --- | --- | --- | --- | --- |
|  |  |  |  |  |  |  |  |
|  |  |  |  |  |  |  |  |
| **جمع** (ريال) | | | | | | |  |

**6-5- هزينه مسافرت (در صورت لزوم):**

| **رديف** | **مقصد** | **تعداد مسافرت در مدت اجراي طرح و منظور آن** | **نوع وسيله نقليه** | **تعداد افراد** | **هزينه**  **(ريال)** |
| --- | --- | --- | --- | --- | --- |
|  |  |  |  |  |  |
|  |  |  |  |  |  |
| **جمع** (ريال) | | | | |  |

**6-6- هزينه هاي تكثير و تايپ:**

| **رديف** | **نوع هزينه** | **هزينه (ريال)** |
| --- | --- | --- |
|  | هزينه هاي تكثير | 000/000/1 |
|  | هزينه هاي تايپ | 000/000/2 |
|  | هزينه سه جلد صحافي گزارش نهايي | 000/000/1 |
| **جمع** (ريال) | | 000/000/4 |

**6-7- ساير هزينه‌ها:**

| **رديف** | **نوع هزينه** | **هزينه (ريال)** |
| --- | --- | --- |
|  | هزينه هاي پيش‌بيني نشده | 000/000/4 |
|  | ساير |  |
| **جمع** (ريال) | | 000/000/4 |

**6-8- جمع هزينه هاي طرح :**

| **رديف** | **نوع هزينه** | **جمع (ريال)** |
| --- | --- | --- |
|  | جمع نيروي انساني(جدول شماره 1) |  |
|  | جمع هزينه هاي آزمايشات و خدمات تخصصي(جدول شماره 2) | 0 |
|  | جمع هزينه هاي وسايل و مواد مصرفي (جدول شماره 3) | 102025000 |
|  | جمع هزينه هاي وسايل و مواد غيرمصرفي (جدول شماره 4) |  |
|  | جمع هزينه هاي مسافرت (جدول شماره 5) |  |
|  | جمع هزينه‌هاي تكثير و تايپ (جدول شماره 6) | 0 |
|  | ساير هزينه ها (جدول شماره 7) | 0 |
| **جمع كل** (ريال) | | 102025000 |

**6-9- هزينه‌هاي طرح به تفكيك سال‌هاي اجرا:**

| **نوع هزينه‌** | **جمع هزينه ها** | | | | | **جمع** |
| --- | --- | --- | --- | --- | --- | --- |
|  | سال اول | سال دوم | سال سوم | سال چهارم | سال پنجم |  |
| هزينه پرسنلي  هزينه‌هاي آزمايشات و خدمات تخصصي  هزينه‌هاي وسايل و مواد  هزينه‌هاي مسافرت  هزينه‌هاي ديگر | **102025000** |  |  |  |  |  |
| **جمع** **کل** (ریال) |  |  |  |  |  | 102025000 |

**7- فرم اظهارنامه پژوهشگر و ارزيابي اخلاق در پژوهش**

اين فرم بايد توسط مجري/مجريان تكميل و همراه با طرح پژوهشي به كميته اخلاق در پژوهش انستيتو تحقيقات تغذيه‌اي و صنايع غذايي كشور ارائه شود.

موضوع پژوهش: تاثیر کوله کلسیفرول، ایزوفلاونهای سویا و تجویز توام انها بر علایم بالینی، کیفیت زندگی، سیتوکین التهابی، ظرفیت تام آنتی اکسیدانی سرم و میزان نفوذپذیری روده در بیماران مبتلا به سندروم روده تحریک پذیر

نام مجري/مجريان: دکتر آزیتا حکمت دوست- مهسا جلیلی

محل اجراي پژوهش: کلینیک بيماريهاي گوارش و کبد دانشگاه تهران

كليه موارد ذيل بايد داراي مدارك و مستندات لازم باشد.

|  | **بله** | **خير** | **نظر كميته اخلاق در پژوهش انستيتو** |
| --- | --- | --- | --- |
| 1- آيا باورها، رفتارها، سنتهاي جامعه و موازين شرعي رعايت شده است؟ | ■ | € | ............................................................ |
| 2- آيا در تمام مراحل عدم وجود مطالب زننده و غير متعارف رعايت شده است؟ | ■ | € | ............................................................ |
| 3- آيا در بازنگري مدارك رعايت صداقت و امانت شده است؟ | ■ | € | ............................................................ |
| 4- آيا از منابع معتبر و مورد تأييد استفاده گرديده است؟ | ■ | € | ............................................................ |
| 5- آيا از جديدترين روش‌هاي پژوهش استفاده شده است؟ | ■ | € | ............................................................ |
| 6- آيا روش‌هاي بكار رفته فاقد اثرات سوء جسمي و روحي هستند؟ | ■ | € | ............................................................ |
| 7- آيا رعايت آزادي فردي آزمودني يا بيمار شده است؟ | ■ | € | ............................................................ |
| 8- آيا فرم رضايت آگاهانه تنظيم شده و به امضاي افراد خواهد رسيد؟  (يك نسخه از فرم مزبور را ضميمه كنيد) | ■ | € | ............................................................ |
| 9- آيا ضرر و زيان ناشي از پژوهش كمتر از منافع آن است؟ | ■ | € | ............................................................ |
| 10- آيا خسارات ناشي از صدمات جسمي يا زيان مالي ناشي از پژوهش كه ممكن است ناخواسته به افراد مورد بررسي وارد شود، جبران خواهد شد؟ | ■ | € | ............................................................ |
| 11- آيا حقوق افراد صغير و يا كساني كه قيم لازم دارند حفظ شده است؟ | ■ | € | ............................................................ |
| 12- آيا قرار گرفتن تصادفي آزمودني در گروه شاهد و استفاده از دارونما به اطلاع وي خواهد رسيد؟ | ■ | € |  |
| 13- آيا با قرار گرفتن آزمودني در گروه شاهد و يا تجويز دارونما، آزمودني كماكان داراي حق بهره‌مندي از خدمات ضروري است؟ | ■ | € | ............................................................ |
| 14- آيا اطلاعات مربوط به افراد مورد بررسي محرمانه باقي خواهد ماند؟ | ■ | € | ............................................................ |
| 15- آيا كليه اهداف و روش‌هاي اجراي پژوهش و نوع مداخله احتمالي از پژوهش به كميته اخلاق در پژوهش اعلام شده است؟ | ■ | € | ............................................................ |
| 16- آيا كليه اطلاعات لازم در خصوص اهداف، روش‌هاي اجراي پژوهش، نوع مداخله احتمالي و نتايج حاصل از آن به اطلاع آزمودني خواهد رسيد؟ | ■ | € | ............................................................ |
| 17- آيا به آزمودني در مورد حق انصراف و خروج از پژوهش آگاهي كافي داده خواهد شد؟ | ■ | € | ............................................................ |

**امضاء مجري/مجريان .................... .................. تاريخ ....20/5/1392...............................**

ــــــــــــــــــــــــــــــــــــــــــــــــــــــــــــــــــــــــــــــــــــــــــــــــــــــــــــــــــــــــــــــ

- نظر نهايي كميته اخلاق در پژوهش انستيتو در مورد رعايت اصول اخلاقي در پژوهش:

در جلسه ........................... مورخ........................ كميته اخلاق مورد تاييد قرار گرفت.

**امضاء دبير كميته اخلاق در پژوهش انستيتو ...................................**

**8- فهرست منابع** (به راهنمای نگارش منابع در صفحه آخر مراجعه شود.)

در پایان پیشنهاد طرح پژوهشی آمده است.

**9- پرسش نامه ها، برگه‌هاي اطلاعاتي و جداول توخالي**

# جداول توخالی

**جدول 1 –میانگین و انحراف معیار TNF-α در بیماران مبتلا به سندروم روده تحریک پذیر**

| شاخص ها | گروه | تعداد | شروع مطالعه | هفته ششم | p-value |
| --- | --- | --- | --- | --- | --- |
| TNF-α (pg/ml) | ایزوفلاون سویا |  |  |  |  |
|  | ویتامین D |  |  |  |  |
|  | ایزوفلاون سویا-ویتامین D |  |  |  |  |
|  | دارونما |  |  |  |  |

**
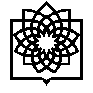
**

دانشگاه علوم پزشكي و خدمات بهداشتي درماني شهيدبهشتي

گروه تغذيه دانشكده علوم تغذيه و صنايع غذايي

**فرم رضایت نامه**

پروژه پژوهشی : تاثیر کوله کلسیفرول، ایزوفلاونهای سویا و تجویز توام انها در مقایسه با دارونما بر علایم بالینی، کیفیت زندگی، سیتوکین التهابی TNF-alpha، ظرفیت تام آنتی اکسیدانی سرم TAC و میزان نفوذپذیری روده در بیماران مبتلا به سندروم روده تحریک پذیر

اینجانب ................................ بدین وسیله موافقت خود را برای شرکت در مطالعه " تاثیر کوله کلسیفرول، ایزوفلاونهای سویا و تجویز توام انها در مقایسه با دارونما بر علایم بالینی، کیفیت زندگی، سیتوکین التهابی TNF-alpha، ظرفیت تام آنتی اکسیدانی سرم TAC و میزان نفوذپذیری روده در بیماران مبتلا به سندروم روده تحریک پذیر " اعلام می دارم. به منظور انجام این مطالعه در 2 تاریخ معین 5 سی سی خون بعد از 14-12 ساعت ناشتایی گرفته خواهد شد و در طی 6 هفته افرادی که در گروه دریافت کننده مکمل ایزوفلاون سویا، ویتامین D و ایزوفلاون سویا-ویتامین D قرار می گیرند باید روزانه دو کپسول ایزوفلاون سویا همراه با دارونمای ویتامین D یا یک کپسول دوهفتگی ویتامین D همراه با دارونمای ایزوفلاون سویا و یا به طور توام دو کپسول روزانه ایزوفلاون سویا و کپسول دوهفتگی ویتامین D را که فاقد عوارض جانبی می باشند دریافت نمایند در حالی که افراد گروه دارونما روزانه دو کپسول دارونمای ایزوفلاون سویا و یک کپسول دوهفتگی ویتامین D دریافت خواهند کرد افراد در طی این مدت از توصیه های رژیمی و ورزشی که رژیم شناس برای فرد تعیین می کند، تبعیت می کند. در این پژوهش کلیه آزمایشات خون رایگان خواهد بود و اطلاعاتی که گرفته می شود محرمانه باقی خواهد ماند . همچنین برای اینجانب این حق محفوظ می باشد که در صورت عدم تمایل ، به همکاری خود در این پروژه خاتمه دهم و در صورت هر گونه ضرر و زیان نیز این موافقت نامه مانع از اقدام قانونی اینجانب در مقابل مجریان این پژوهش و دانشگاه نخواهد شد. شرکت داوطلبانه در این پژوهش می تواند در ایجاد روش های پیشگیری از پیشرفت سندروم روده تحریک پذیر در این بیماران مؤثر باشد.

آدرس بیمار شرکت کننده در پژوهش : ...........................................................................................................

تلفن منزل : ................................................... تلفن محل کار : .......................................................................

تاریخ : ................................... امضاء .....................................................


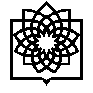


دانشگاه علوم پزشكي و خدمات بهداشتي درماني شهيدبهشتي

گروه تغذيه دانشكده علوم تغذيه و صنايع غذايي

**فرم جمع آوری داده ها**

پروژه پژوهشی : تاثیر کوله کلسیفرول، ایزوفلاونهای سویا و تجویز توام انها در مقایسه با دارونما بر علایم بالینی، کیفیت زندگی، سیتوکین التهابی TNF-alpha، ظرفیت تام آنتی اکسیدانی سرم TAC و میزان نفوذپذیری روده در بیماران مبتلا به سندروم روده تحریک پذیر

نام و نام خانوادگی بیمار : ..................................................... تاریخ ورود به مطالعه : ............................. تاریخ مراجعه پایانی : ........................

تلفن : ................................................ آدرس بیمار : ..............................................................................................................................

استعمال سیگار : بلی خیر

یائسگی:

دریافت روغن ماهی: مقدار : شکل:

**مصرف داروهای زیر در طی یک ماه گذشته :**

مکمل های ایزوفلاون سویا بلی خیر داروهای ضد التهابی بلی خیر

مکمل ویتامین D بلی خیر داروهای آنتی بیوتیکی بلی خیر

داروهای تغییر حرکت گوارشی بلی خیر شیرین کننده های مصنوعی بلی خیر

داروهای خونریزی دهنده گوارشی بلی خیر نیکوتین و مواد مخدر بلی خیر

داروهای مسهل و ضد اسهال بلی خیر داروهای سرکوب کننده ایمنی بلی خیر

**ابتلا به بیماری های :**

جراحی عمده کولون بلی خیر بیماری های ارگانیک روده بلی خیر اختلال شدید روانی بلی خیر

بارداری یا شیردهی بلی خیر ورزشکار بلی خیر

در صورتیکه بیمار حتی یکی از داروهای ذکر شده را در طی یک ماه گذشته مصرف کرده باشد و یا به یکی از بیماری های نامبرده مبتلا باشد وارد مطالعه نخواهد شد.

**مواجهه با نور آفتاب:**

- مدت زمان مواجهه با نور آفتاب تقریبا چه مدت در روز است؟

در مقابل آفتاب قرار نمی گیرد 10 دقیقه تا یک ساعت 2-1 ساعت بیشتر از 2 ساعت

- ساعات مواجهه شما با نور آفتاب معمولا چه موقعی از روز و چه مدت است؟ لطفا ساعت ذکر شود

ساعت 10-7 چند دقیقه 15-10 چند دقیقه 17-15 چند دقیقه

- معمولا در زمان مواجهه با نور آفتاب چه قسمتهایی از بدن بدون پوشش در برابر نور قرار می گیرند؟

صورت دست از مچ به پایین پاها دست از بازو به پایین

- آیا از کرم ضد آفتاب در مواجهه با نور آفتاب استفاده می کنید؟

خیر گاهی اغلب

_

| ردیف | نام متغیر | کد / اندازه متغیر | | ردیف | نام متغیر | اندازه متغیر | |
| --- | --- | --- | --- | --- | --- | --- | --- |
|  |  |  |  |  |  | شروع مطالعه | پایان مطالعه |
| 1 | گروه درمانی |  | | 7 | میزان مانیتول ادراری |  |  |
| 2 | سن (سال) |  | | 8 | میزان لاکتولوز ادراری |  |  |
| 3 | قد (سانتیمتر) |  | | 9 | نسبت مانیتول به لاکتولوز ادراری |  |  |
| 4 | تعداد مصرف روزانه سیگار |  | | 10 | 25-هیدروکسی ویتامین D سرمی |  |  |
| نام متغیر | | شروع مطالعه | پایان مطالعه | 11 | TNF-α سرم (pg/dL) |  |  |
| 5 | وزن (kg) |  |  | 12 | TAC (mMol)سرمی |  |  |
| 6 | BMI (kg/m^2^) |  |  |  |  |  |  |

**
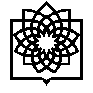
**

دانشگاه علوم پزشكي و خدمات بهداشتي درماني شهيدبهشتي

گروه تغذيه دانشكده علوم تغذيه و صنايع غذايي

پرسشنامه یادآمد 24 ساعته خوراک

پروژه پژوهشی : تاثیر کوله کلسیفرول، ایزوفلاونهای سویا و تجویز توام انها در مقایسه با دارونما بر علایم بالینی، کیفیت زندگی، سیتوکین التهابی TNF-alpha، ظرفیت تام آنتی اکسیدانی سرم TAC و میزان نفوذپذیری روده در بیماران مبتلا به سندروم روده تحریک پذیر

نام و نام خانوادگی:

| وعده غذایی | نوع غذاواجزاء تشکیل دهنده | مقدار مصرف | تبدیل به گرم | ملاحظات |
| --- | --- | --- | --- | --- |
| صبحانه |  |  |  |  |
| میان وعده |  |  |  |  |
| ناهار |  |  |  |  |
| میان وعده عصر |  |  |  |  |
| شام |  |  |  |  |
| قبل از خواب |  |  |  |  |

نام ونام خانوادگی پرسشگر: تاریخ: امضا

**پرسشنامه کیفیت زندگی سندرم روده تحریک پذیر (IBS-QOL)**

**توضیحات:** هدف سؤالات زیر بررسی میزان تأثیر **سندرم روده تحریک پذیر** یا **آی بی اِس** بر کیفیت زندگی شما و همچنین بررسی مشکلات احتمالی است که این بیماری برای شما ایجاد کرده است. لطفاً در مورد زندگی خودتان **در طی یک ماه گذشته (30 روز گذشته)** فکر کنید، و به سؤالات 1 تا 34 پاسخ دهید. هر سؤال پنج پاسخ متفاوت دارد که بسته به شدت علایم و مشکلات ایجاد شده می توانید **یکی** از گزینه های "**به هيچ وجه**" ، "**كمي**" ، "**نسبتاً**" ، "**زياد**" یا "**به شدت زیاد**" را در هر سؤال انتخاب کنید. برای هر سؤال، لطفاً پاسخی که به بهترین نحو احساس شما را بیان می کند، به صورت **×** در مربع علامت بزنید و به همه سؤالات پاسخ دهید.

1. به دلیل مشكلات روده ایم درمانده شده ام.

به هيچ وجه كمي نسبتاً  زياد به شدت زیاد

1. از بوي بد ناشي از مشکلات روده ایم خجالت می کشم.

به هيچ وجه كمي نسبتاً  زياد به شدت زیاد

1. از اینکه برای دفع مدفوع باید خیلی در دستشویی بمانم ناراحت هستم.

به هيچ وجه كمي نسبتاً  زياد بسيار زياد

1. احساس می کنم به خاطر مشكلات روده ایم، در برابر بيماري های دیگر نیز آسيب پذير هستم.

به هيچ وجه كمي نسبتاً  زياد به شدت زیاد

1. احساس مي كنم به خاطر مشكلات روده ایم، مانند افراد چاق شده ام.

به هيچ وجه كمي نسبتاً  زياد بسيار زياد

1. احساس می کنم به دلیل مشكلات روده ایم، دارم كنترل زندگي خود را از دست می دهم.

به هيچ وجه كمي نسبتاً  زياد بسيار زياد

1. به خاطر مشکلات روده ایم، در زندگی ام لذت کمتری احساس می کنم.

به هيچ وجه كمي نسبتاً  زياد بسيار زياد

1. وقتی در مورد مشکلات روده ایم صحبت می کنم، ناراحت می شوم.

به هيچ وجه كمي نسبتاً  زياد به شدت زیاد

1. به خاطر مشكلات روده ایم، احساس افسردگي مي كنم.

به هيچ وجه كمي نسبتاً  زياد به شدت زیاد

1. احساس مي كنم به خاطر مشكلات روده ایم، تنها و منزوی شده ام.

به هيچ وجه كمي نسبتاً  زياد به شدت زیاد

1. به خاطر مشكلات روده ایم، بايد مواظب مقدار غذايي كه مي خورم باشم.

به هيچ وجه كمي نسبتاً  زياد بسيار زياد

1. به خاطر مشكلات روده ایم، انجام فعاليت جنسي برايم مشكل شده است.

به هيچ وجه كمي نسبتاً  زياد به شدت زیاد

1. از اينكه مشكل روده اي دارم، احساس عصبانيت می کنم.

به هيچ وجه كمي نسبتاً  زياد به شدت زیاد

1. حس می کنم به خاطر مشكلات روده ایم، سبب رنجش ديگران شده ام.

به هيچ وجه كمي نسبتاً  زياد بسيار زياد

1. از اينكه مشكلات روده اي من بدتر از این شود نگرانم.

به هيچ وجه كمي نسبتاً  زياد بسيار زياد

1. به خاطر مشكلات روده ایم حساس و زود رنج شده ام.

به هيچ وجه كمي نسبتاً  زياد به شدت زیاد

1. از اینکه مردم فکر کنند در مورد مشکلات روده ایم اغراق می کنم نگرانم.

به هيچ وجه كمي نسبتاً  زياد بسيار زياد

1. احساس مي كنم به خاطر مشكلات روده ایم، كارآيي من كمتر شده است.

به هيچ وجه كمي نسبتاً  زياد بسيار زياد

1. به خاطر مشكلات روده ایم، بايد از موقعيت هاي پر استرس دوری كنم.

به هيچ وجه كمي نسبتاً  زياد بسيار زياد

1. مشكلات روده ایم ميل جنسي ام را كم کرده است.

به هيچ وجه كمي نسبتاً  زياد بسيار زياد

1. به خاطر مشكلات روده ایم، نمي توانم هر نوع لباسي را بپوشم.

به هيچ وجه كمي نسبتاً  زياد بسيار زياد

1. به دلیل مشكلات روده ایم، بايد از انجام كار سنگين پرهيزكنم.

به هيچ وجه كمي نسبتاً  زياد بسيار زياد

1. به خاطر مشكلات روده ایم، احساس مي كنم كه كند و كم تحرك شده ام.

به هيچ وجه كمي نسبتاً  زياد بسيار زياد

1. به خاطر مشكلات روده ایم، بايد مراقب نوع غذايي كه مي خورم باشم.

به هيچ وجه كمي نسبتاً  زياد بسيار زياد

1. به خاطر مشكلات روده ایم، حضور در كنار افرادي كه خوب نمي شناسم برايم دشوار شده است.

به هيچ وجه كمي نسبتاً  زياد بسيار زياد

1. به دلیل مشكلات روده ایم، احساس مي كنم تمیز نیستم.

به هيچ وجه كمي نسبتاً  زياد به شدت زیاد

1. به خاطر مشكلات روده ایم، رفتن به مسافرت هاي طولاني برايم مشکل شده است.

به هيچ وجه كمي نسبتاً  زياد به شدت زیاد

1. از اينكه به خاطر مشكلات روده ایم نمي توانم هر وقت دوست دارم غذا بخورم، احساس کمبود می کنم.

به هيچ وجه كمي نسبتاً  زياد به شدت زیاد

1. به خاطر مشكلات روده ایم، نزدیک بودن به دستشويي برایم مهم است.

به هيچ وجه كمي نسبتاً  زياد به شدت زیاد

1. زندگی ام حول مشکلات روده ایم می چرخد.

به هيچ وجه كمي نسبتاً  زياد بسيار زياد

1. نگران هستم كه اختیار دفع مدفوع را از دست بدهم.

به هيچ وجه كمي نسبتاً  زياد بسيار زياد

1. از اینکه نتوانم مدفوعم را دفع کنم، نگرانم هستم.

به هيچ وجه كمي نسبتاً  زياد بسيار زياد

1. مشكلات روده ایم، نزديك ترين روابطم را تحت تاثير قرار داده است.

به هيچ وجه كمي نسبتاً  زياد بسيار زياد

1. احساس مي كنم که هيچ كس مشكلات روده ای من را درك نمي كند.

به هيچ وجه كمي نسبتاً  زياد به شدت زیاد

**پرسشنامه طرح** " تاثیر کوله کلسیفرول، ایزوفلاونهای سویا و تجویز توام انها در مقایسه با دارونما بر علایم بالینی، کیفیت زندگی، سیتوکین التهابی TNF-alpha، ظرفیت تام آنتی اکسیدانی سرم TAC و میزان نفوذپذیری روده در بیماران مبتلا به سندروم روده تحریک پذیر **"**

نام و نام خانوادگي: تاريخ مراجعه: کد بيمار: تاريخ تولد:

سن بيمار: وضعيت تاهل: شغل: تحصيلات:

محل سکونت: شماره تلفن:

**1**. درد يا ناراحتي شکم:

**بدون درد کمی آزاردهنده ناخوشايند درد شديد درد بسيار شديد غير قابل تحمل**

2. نفخ يا احساس اتساع شکم:

**بدون نفخ کمی آزاردهنده ناخوشايند نفخ شديد نفخ بسيار شديد غير قابل تحمل**

**3**. احساس سريع به اجابت مزاج

**بدون احساس فوريت کمی آزاردهنده ناخوشايند فوريت شديد فوريت بسيار شديد غير قابل تحمل**

**4**. تعداد دفعات اجابت مزاج روزانه**: □**

**5. لطفا ﹰ شکل مدفوع خود را در اشکال زير علامت بزنيد:**

**
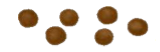

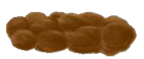

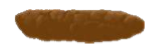

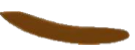

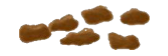

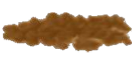

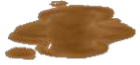
**  7 . □ 6. □ 5. □ 4. □ 3. □ 2. □ 1. □

**6. چگونگي دفع خود را توصيف نماييد.** 1 **. خارج کردن مدفوع به کمک دست 2. خارج کردن مدفوع به کمک تنقيه 3. خارج کردن مدفوع به کمک زور زدن 4.تخليه طبيعي 5. نياز به تخليه فوري بدون درد شکم 6. نياز به تخليه فوري همراه با درد شکم 7. بي اختياري در دفع**

**7. آيا شما احساس دغع ناکامل داشته ايد؟ 1.خير □ 2. بله □**

**8. آيا به دنبال مصرف داروها دچار مشکل خاصی شده ايد؟ اگر ايجاد شده است نوع و شدت آن را ذکر نماييد :**

1**. خير □ 2. بله□ نوع مشکل ايجاد شده : ..........................................................**

**1-2) کم □ 2-2) متوسط 3-2) شديد □**

9. آيا پس از 7 روز مصرف دارو احساس رضايت می نماييد؟ **1.خير □ 2. بله □**

**
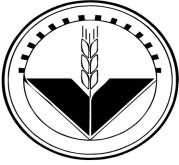
**

**انستيتو تحقيقات تغذيه‌اي و صنايع غذايي كشور**

**معاونت پژوهشي - كميته اخلاق در پژوهش**

**اظهارنامه خون‌گيري در بزرگسالان**

**عنوان طرح تحقيقاتي:** تاثیر کوله کلسیفرول، ایزوفلاونهای سویا و تجویز توام انها در مقایسه با دارونما بر علایم بالینی، کیفیت زندگی، سیتوکین التهابی TNF-alpha، ظرفیت تام آنتی اکسیدانی سرم TAC و میزان نفوذپذیری روده در بیماران مبتلا به سندروم روده تحریک پذیر

**مجري مسئول:** دکتر آزیتا حکمت دوست، مهسا جلیلی

1. آيا نمونه خون در طي يك نمونه‌گيري روتين تشخيصي - درماني انجام مي‌گيرد يا گرفتن نمونه خون تنها به منظور تحقيق مورد نظر مي‌باشد؟

گرفتن نمونه خون صرفا به منظور تحقیق می باشد.

1. ميزان نمونه خون مورد نياز به چه اندازه است؟

5 سی سی خون وریدی قبل و بعد از مداخله

1. آيا در مورد اقداماتي كه بر روي نمونه انجام مي‌شود به فرد و يا قيم قانوني او توضيح داده مي‌شود؟ با ذكر توضيحات.

بله، مراحل نمونه گیری از بیمار و آزمایشهایی که بر روی نمونه ها انجام خواهد گرفت به بیمار توضیح داده می شود و در صورت ابراز رضایت آگاهانه در مطالعه شرکت داده خواهد شد.

1. در چه مواردي روند خون‌گيري از فرد متوقف خواهد شد؟

در صورتی که فرد دچار عوارض حین خونگیری از جمله افت ناگهانی فشارخون یا عدم پیداکردن رگ مناسب و یا هر عارضه ای که به تشخیص پزشک محقق و یا مخالفت فرد شرکت کننده در اثر خونگیری ایجاد شده باشد موجب توقف خونگیری خواهد شد.

1. در صورت امتناع فرد يا قيم وي از نمونه‌گيري، چه نوع تمهيداتي براي جايگزين كردن فرد ديگر انديشيده شده است؟

در صورت امتناع فرد از خونگیری، این فرد جزء افراد ترک کننده مطالعه محسوب شده و علت خروج وی از مطالعه نیز در فرم ثبت بیماران ذکر خواهد شد و فرد دیگر واجد معیارهای مطالعه جایگزین خواهد شد.

1. مكان خون‌گيري داراي كدام تجهيزات و وسايل لازم براي خون‌گيري است؟

خونگیری در کلینیک تغذیه دانشکده تغذیه علوم پزشکی شهید بهشتی انجام خواهد شد که دارای کارشناس مجرب و امکانات خونگیری می باشد.

1. آيا در اين مكان كاركنان آموزش ديده و امكانات لازم براي كنترل موارد اورژانس و احيا مريض وجود دارد؟ (به تفكيك نام ببريد.)

بله، کلینیک مزبور دارای کادر پزشک برای کنترل موارد اورژانس و نیز در صورت وخامت اوضاع، امکان ارجاع به مراکز اورژانس دانشگاه علوم پزشکی شهید بهشتی وجود دارد.

1. خون‌گيري توسط چه كسي و با چه ميزان مهارت انجام خواهد شد؟ (مشخصات فردي، تحصيلات و سابقه ذكر شود.)

خون گیری توسط کارشناس مجرب و آموزش دیده صورت خواهد گرفت که ایشان سابقه همکاری در چندین طرح پژوهشی دانشکده تغذیه شهید بهشتی را هم داشته اند.

9. مسئوليت فرايند خون‌گيري و عواقب ناشي از آن بر عهده چه كسي خواهد بود؟

مجریان طرح مسئول فرآیند خونگیری و عواقب آن هستند.

10. با توجه به نمونه مورد نياز خون‌گيري توسط كدام يك از روش‌هاي زير انجام خواهد شد؟

 Finger Prick

Venous sampling:  Butterfly Needle ■ Syringe

Peripheral Arterial

Paper

Surgical long lines

Percutaneous Long Lines

Femoral Line

Angiocat

11. در صورت نمونه‌گيري از وريد، آيا از بي حسي موضعي براي كنترل درد استفاده مي‌شود؟

خیر، افراد شرکت کننده بالغین بالای 18 سال هستند که نیازی به بی حسی موضعی وجود ندارد.

12. در نمونه‌گيري از وريد محيطي از كدام يك از وريدهاي زير نمونه گرفته خواهد شد؟

آرنج■

پشت دست

وريدهاي پا

13. آيا لازم است فرد در هنگام خون‌گيري ناشتا باشد؟ در صورت مثبت بودن پاسخ براي چند ساعت؟

بله، فرد شرکت کننده باید بین 10-12 ساعت ناشتا باشد

14. آيا به فرد پس از خون‌گيري ميان وعده غذائي داده مي‌شود؟ اگر بلي شامل چه مواردي؟

بله، شامل یک کیک صبحانه ساده با یک پاکت آبمیوه

| **نام و امضا مجري** | **نام و امضا فرد مسئول خون‌گيري** |
| --- | --- |

**10- نكات مهم**

1. اينجانب دکتر آزیتا حکمت دوست به عنوان مجري مسئول طرح، با آگاهي كامل از مفاد دستورالعمل پژوهشي انستيتو، مسئوليت كليه مراحل اجرايي، قانوني و حقوقي، اداري و مالي طرح را مي‌پذيرم و نشاني خود را برای انجام تمامي مكاتبات اداري و مالي طرح به این شرح اعلام می‌کنم:

تهران، میدان صنعت، بلوار فرحزادی، ارغوان غربی، دانشکده علوم تغذیه و صنایع غذایی شهید بهشتی، گروه تغذیه بالینی و رژیم درمانی. تلفن: 5- 22357483- داخلی: 239

1. آدرس نويسنده اول يا نويسنده مسئول در تمامي مقاله حاصل از طرح پژوهشي در مقاله‌‌هاي فارسي و انگليسي بايد به شكل زير باشد:
2. آدرس فارسي: گروه تغذیه بالینی و رژیم درمانی، انستيتو تحقيقات تغذيه‌اي و صنايع غذايي كشور، دانشكده علوم تغذيه و صنايع غذايي، دانشگاه علوم پزشكي شهيد بهشتي.

**نشاني انگليسي:**

Clinical Nutrition and Diet Therapy, National Nutrition and Food Technology Research Institute, Faculty of Nutrition Sciences and Food Technology, Shahid Beheshti University of Medical Sciences, Tehran, Iran.

1. يك نسخه از مقاله حاصل از طرح، قبل از submission جهت كنترل موارد مورد نظر انستيتو به معاونت پژوهشي تحويل گردد.
2. يك نسخه از مقاله، پس از چاپ جهت حفظ در سوابق، به معاونت پژوهشي ارائه گردد (حتي پس از اتمام مدت اجراي قرارداد و اخذ تسويه حساب نهايي).
3. بر اساس دستورالعمل وزارت بهداشت چنانچه طرح تحقيقاتي، پايان نامه نيز باشد، در متن مقاله حاصله، اين موضوع تصريح شود.
4. جمله زير در متن مقاله، حتما بايد ذكر شود :

«اين مقاله حاصل طرح پژوهشي " تاثیر کوله کلسیفرول، ایزوفلاونهای سویا و تجویز توام انها در مقایسه با دارونما بر علایم بالینی، کیفیت زندگی، سیتوکین التهابی TNF-alpha، ظرفیت تام آنتی اکسیدانی سرم TAC و میزان نفوذپذیری روده در بیماران مبتلا به سندروم روده تحریک پذیر " مي‌باشد كه انستيتو تحقيقات تغذيه‌اي و صنايع غذايي كشور بودجه اجراي آن را تأمين كرده است.»

1. ثبت كارآزمائي باليني در سايت <http://www.irct.ir/fa> قبل از درخواست پيش پرداخت اول انجام شود.

9- پرداخت حداكثر 25 درصد از حق‌التحقيق طرح به عنوان حسن انجام كار منوط به احراز شرايط زير مي‌باشد:

الف ـ اتمام به موقع طرح: تمديد مدت اجراي طرح، منوط به موافقت شوراي پژوهشي بوده و با تشخیص آن شورا منجر به كسر ده درصد از مبلغ حسن انجام كار مي‌شود.

ب ـ نمره گزارش نهايي: در صورت كسب نمره متوسط، 5 درصد و نمره ضعيف 10 درصد از ميزان حسن انجام كار كسر خواهد شد.

نام و امضای مجري مسئول: دکتر آزیتا حکمت دوست

|  |  |
| --- | --- |
| نام و امضاي مجریان به همراه درج **آدرس/شماره شناسنامه/صادره از/کد ملی**:   \| 1. دکتر آزیتا حکمت دوست..... تهران، میدان صنعت، بلوار فرحزادی، ارغوان غربی، دانشکده علوم تغذیه و صنایع غذایی شهید بهشتی، گروه تغذیه بالینی و رژیم درمانی. تلفن: 5- 22357483- داخلی: 239   شماره شناسنامه 5496  کد ملی 0064072551   1. مهسا جلیلی تهران، میدان صنعت، بلوار فرحزادی، ارغوان غربی، دانشکده علوم تغذیه و صنایع غذایی شهید بهشتی، گروه تغذیه بالینی و رژیم درمانی. تلفن: 5- 22357483- داخلی: 239 . \|  \| \| --- \| --- \| \| شماره شناسنامه 6393  کد ملی 1382551967 \|  \| |  |

**برگه اطلاعاتی طرح پژوهشي**

عنوان و كد طرح موردنظر: تاثیر کوله کلسیفرول، ایزوفلاونهای سویا و تجویز توام انها در مقایسه با دارونما بر علایم بالینی، کیفیت زندگی، سیتوکین التهابی، ظرفیت تام آنتی اکسیدانی سرم و میزان نفوذپذیری روده در بیماران مبتلا به سندروم روده تحریک پذیر می باشد.

نام سازمان مسئول اجراي طرح: دانشگاه علوم پزشکی شهید بهشتی می باشد.

نام سازمان پشتيبان اجراي طرح: مرکز تحقیقات بیماریهای گوارش دانشگاه علوم پزشکی تهران می باشد.

عنوان و هدف از اجراي طرح به زبان ساده: تاثیر ویتامین D، ماده موثره سویا و تجویز توام انها بر علایم بالینی، کیفیت زندگی، یکی از فاکتورهای التهابی، ظرفیت تام آنتی اکسیدانی سرم و میزان نفوذپذیری روده در بیماران مبتلا به سندروم روده تحریک پذیر می باشد.

اهداف از انجام این طرح: الف – تعیین جنس ، قد و سن در شروع مطالعه و وزن، BMI، اجزای رژیم غذایی، TNF-α و TAC ، علایم بالینی، کیفیت زندگی در شروع و پایان مطالعه در بیماران 4 گروه دریافت کننده مکمل ایزوفلاون سویا، ویتامین D ، ویتامین D- سویا توام و گروه دارونما.

ب – مقایسه شاخص آنتی اکسیدانی و شاخص التهابی در شروع و پایان مطالعه در بیماران 4 گروه دریافت کننده مکمل ایزوفلاون سویا، ویتامین D ، ویتامین D- سویا توام و گروه دارونما.

ج- مقایسه میانگین غلظت ادراری لاکتولوز و مانیتول و نسبت آنها در شروع و پایان مطالعه در بیماران 4 گروه دریافت کننده مکمل ایزوفلاون سویا، ویتامین D ، ویتامین D- سویا توام و گروه دارونما می باشند.

در این مطالعه از شما در صورت تمایل برای شرکت در این تحقیق ، پس از 12 تا 14 ساعت ناشتایی ، 5 سی سی خون گرفته می شود و سرم خون تان جهت اندازه گیری غلظت سیتوکین التهابی TNF-alphaدر فریزر نگهداری می شوند. در شروع مطالعه، ابتدا اهداف و روش اجرای مطالعه برای شما توضیح داده می شود و سپس رضایت نامه کتبی گرفته می شود. شما به روش بلوکه بندی شده تصادفی متعادل شده به 4 گروه دریافت کننده مکمل ایزوفلاون سویا، ویتامین D ، ایزوفلاون سویا-ویتامین D یا گروه دارو نما تقسیم می شوید. همچنین در شروع مطالعه وزن، قد شما اندازه گیری می شوند و BMI با استفاده از فرمول (تقسیم وزن به کیلوگرم بر مجذور قد به مترمربع) بدست خواهد آمد. برگه مشخصات عمومی برای شما تکمیل می گردد و نیز پرسشنامه معتبر علایم بالینی و کیفیت زندگی در ابتدای مطالعه و پایان هفته ششم تکمیل می شود. در این مطالعه به منظور بررسی رژیم غذایی شما، در ابتدای مطالعه و پایان هفته ششم از شما سه روز یادآمد خوراک 24 ساعته در مورد یک روز تعطیل و دو روز غیر تعطیل، از طریق مصاحبه حضوری و تلفنی تکمیل می­شود.

در این مطالعه شما برحسب گروهی که در آن قرار می گیرند به مدت 6 هفته مکمل های مربوطه را دریافت می نمایید. شما در گروه دریافت کننده مکمل ایزوفلاون سویا ، روزانه دو کپسول حاوی 20 میلی گرم ایزوفلاون سویا (در مجموع 40 میلی گرم) همراه با دارونمای دوهفته ای یکبار ویتامین D دریافت خواهید کرد، همچنین شما درگروه دریافت کننده مکمل ویتامین D به میزان 50000 واحد در دوهفتگی همراه با دارونمای ایزوفلاون سویا دریافت خواهید کرد و شما در گروه دریافت کننده مکمل ایزوفلاون سویا توام با ویتامین D مکمل روزانه ایزوفلاون سویا با مکمل دوهفتگی ویتامین D دریافت خواهید کرد در حالی که به شما در گروه دارونما روزانه دو کپسول دارونمای ایزوفلاون سویا و یک دارونمای دوهفتگی ویتامین D داده می شود که این کپسول ها از نظر ظاهری، مشابه کپسول مکمل همنام خود می­باشد. قوطی­های حاوی کپسول­ در شروع مطالعه به تعداد کافی به شما داده می­شود و از شما خواسته می­شود هر روز دو عدد کپسول مکمل یا دارونمای ایزوفلاون سویا و نیز دوهفتگی یکبارکپسول مکمل با دارونمای ویتامین D مصرف نمایید. در پایان مطالعه با اندازه گیری مجدد وزن شما ، BMI شما محاسبه اندازه گیری می­شود و از شما پس از 12 تا 14 ساعت ناشتایی 5 سی سی نمونه خون گرفته می­شود. میزان نفوذپذیری روده به روش میزان دفع ادراری مانیتول و لاکتولوز (بعد از خوردن 5 گرم بر دسی لیتر لاکتولوز و 1 گرم بر دسی لیتر مانیتول) اندازه گيري مي شود. همچنین، سطح سرمی 25-هیدروکسی ویتامین D در ابتدای مطالعه و پایان هفته ششم به منظور تعیین وضعیت پایه ویتامین D و نیز ارزیابی میزان مصرف کپسولها اندازه گیری می شود و از پرسشنامه تعیین دریافت ویتامین D از نورخورشید نیز استفاده می شود.

پیگیری شما در این پژوهش، به منظور کنترل آن­ها از نظر مصرف کپسول­ها و جلوگیری از ریزش نمونه­ها تقریبا هر هفته یک بار بصورت تلفنی انجام خواهد شد و در پایان هفته ششم نیز با شمارش کپسول­های باقیمانده میزان رعایت شما از نظر مصرف کپسول­ها مورد ارزشیابی قرار می­گیرد و اگر شما که بیش از 10 درصد کپسول­های خود را مصرف نکرده باشید از تحقیق کنار گذاشته می­شوید. همچنین تغییر عمده در رژیم غذایی یا فعالیت فیزیکی در طول مطالعه موجب حذف از مطالعه می شود.

بر اساس مطالعات پیشین، مکملهای ویتامین D و سویا عوارض جانبی گزارش شده ندارند و در صورت مشاهده هرگونه عارضه جانبی شما با آزادی تمام، حق خروج از مطالعه را دارید.

تمام اطلاعات مربوط به شما محرمانه و در نزد پژوهشگران به شکل کدبندی شده نگهداری می شود و تصمیم بر عدم شرکت در مطالعه هیچ تاثیری بر دریافت درمانهای معمول مورد نیاز ندارد.

عدم شرکت شما در مطالعه کاملا آزاد بوده و شما در هر زمان که بخواهید می توانید بدون هیچ محرومیتی مطالعه را ترک کنید و از درمانهای معمول بهره مند شوید.

شما آزاد هستید تا در هر زمان که بخواهید از مطالعه خارج شوید و هیچ عواقبی برای شما نخواهد داشت.

اگر نتایج حاصل از مطالعه موجب حفظ و ارتقای سلامت افراد بیمار شود در اختیار شما و یا سایر افراد قرار داده خواهد شد ولی مداخله به منظور درمان یا پیشگیری از سندروم روده تحریک پذیر به عهده پژوهشگر این طرح تحقیقاتی نیست.

تمام هزینه های مربوط به انجام طرح تحقیقاتی بر عهده پژوهشگرین این طرح است و شما به طور رایگان از خدمات این طرح بهره مند می شوید.

در صورتی که خسارتهای احتمالی ناشی از این طرح تحقیقاتی بر شما متحمل گردد، جبران آنها بنا به مقررات و بر عهده پژوهشگران خواهد بود.

در صورت تایید اثرات مثبت مداخلات مورد پژوهش یک راهکار ارزانتر, ایمن تر در اختیار شما قرار خواهد گرفت.

دکتر آزیتا حکمت دوست مجری اصلی طرح پزشک متخصص تغذیه عضو هیات علمی دانشکده تغذیه شهید بهشتی تهران، میدان صنعت، بلوار فرحزادی، ارغوان غربی، دانشکده علوم تغذیه و صنایع غذایی شهید بهشتی، گروه تغذیه بالینی و رژیم درمانی. تلفن: 5- 22357483- داخلی: 239

امضا تاریخ

مهسا جلیلی دانشجوی دکترای تخصصی تغذیه دانشکده تغذیه شهید بهشتی

امضا تاریخ

نام افراد مسئول پاسخگويي به سوالات: مهسا جلیلی

تهران، میدان صنعت، بلوار فرحزادی، ارغوان غربی، دانشکده علوم تغذیه و صنایع غذایی شهید بهشتی، گروه تغذیه بالینی و رژیم درمانی. 09396708282

**پیوست گزارش GPOWER به منظور محاسبه حجم نمونه**

[1] -- Monday, August 26, 2013 -- 07:13:25

t tests - Means: Difference between two independent means (two groups)

Analysis: A priori: Compute required sample size

Input: Tail(s) = Two

Effect size d = 0.8937990

α err prob = 0.05

Power (1-β err prob) = 0.80

Allocation ratio N2/N1 = 1

Output: Noncentrality parameter δ = 2.8962398

Critical t = 2.0210754

Df = 40

Sample size group 1 = 21

Sample size group 2 = 21

Total sample size = 42

Actual power = 0.8067851

**منابع:**

1. Thompson WG, Longstreth GF, Drossman DA, Heaton KW, Irvine EJ, Muller-Lissner SA. Functional bowel disorders and functional abdominal pain. Gut. 1999;45 Suppl 2:II43-7. Epub 1999/08/24.

2. Ritchie J. Pain from distension of the pelvic colon by inflating a balloon in the irritable colon syndrome. Gut. 1973;14(2):125-32. Epub 1973/02/01.

3. Spiller RC, Jenkins D, Thornley JP, Hebden JM, Wright T, Skinner M, et al. Increased rectal mucosal enteroendocrine cells, T lymphocytes, and increased gut permeability following acute Campylobacter enteritis and in post-dysenteric irritable bowel syndrome. Gut. 2000;47(6):804-11. Epub 2000/11/15.

4. Ford AC, Talley NJ. Irritable bowel syndrome. BMJ (Clinical research ed). 2012;345:e5836. Epub 2012/09/07.

5. Karaahmet F, Basar O, Yksel I, Coban S, Yuksel O. Letter: vitamin D supplementation and the irritable bowel syndrome. Alimentary pharmacology & therapeutics. 2013;37(4):499. Epub 2013/01/23.

6. Spiller R, Aziz Q, Creed F, Emmanuel A, Houghton L, Hungin P, et al. Guidelines on the irritable bowel syndrome: mechanisms and practical management. Gut. 2007;56(12):1770-98. Epub 2007/05/10.

7. Stobaugh DJ, Deepak P, Ehrenpreis ED. Increased risk of osteoporosis-related fractures in patients with irritable bowel syndrome. Osteoporosis international : a journal established as result of cooperation between the European Foundation for Osteoporosis and the National Osteoporosis Foundation of the USA. 2013;24(4):1169-75. Epub 2012/09/21.

8. Osterberg E, Blomquist L, Krakau I, Weinryb RM, Asberg M, Hultcrantz R. A population study on irritable bowel syndrome and mental health. Scandinavian journal of gastroenterology. 2000;35(3):264-8. Epub 2000/04/15.

9. Thompson WG, Heaton KW, Smyth GT, Smyth C. Irritable bowel syndrome in general practice: prevalence, characteristics, and referral. Gut. 2000;46(1):78-82. Epub 1999/12/22.

10. Houghton LA, Lea R, Jackson N, Whorwell PJ. The menstrual cycle affects rectal sensitivity in patients with irritable bowel syndrome but not healthy volunteers. Gut. 2002;50(4):471-4. Epub 2002/03/13.

11. Braniste V, Leveque M, Buisson-Brenac C, Bueno L, Fioramonti J, Houdeau E. Oestradiol decreases colonic permeability through oestrogen receptor beta-mediated up-regulation of occludin and junctional adhesion molecule-A in epithelial cells. The Journal of physiology. 2009;587(Pt 13):3317-28. Epub 2009/05/13.

12. Zhou Q, Zhang B, Verne GN. Intestinal membrane permeability and hypersensitivity in the irritable bowel syndrome. Pain. 2009;146(1-2):41-6. Epub 2009/07/15.

13. Gecse K, Roka R, Ferrier L, Leveque M, Eutamene H, Cartier C, et al. Increased faecal serine protease activity in diarrhoeic IBS patients: a colonic lumenal factor impairing colonic permeability and sensitivity. Gut. 2008;57(5):591-9. Epub 2008/01/16.

14. Wada-Hiraike O, Imamov O, Hiraike H, Hultenby K, Schwend T, Omoto Y, et al. Role of estrogen receptor beta in colonic epithelium. Proceedings of the National Academy of Sciences of the United States of America. 2006;103(8):2959-64. Epub 2006/02/16.

15. Morito K, Hirose T, Kinjo J, Hirakawa T, Okawa M, Nohara T, et al. Interaction of phytoestrogens with estrogen receptors alpha and beta. Biological & pharmaceutical bulletin. 2001;24(4):351-6. Epub 2001/04/18.

16. Setchell KD, Brown NM, Zimmer-Nechemias L, Brashear WT, Wolfe BE, Kirschner AS, et al. Evidence for lack of absorption of soy isoflavone glycosides in humans, supporting the crucial role of intestinal metabolism for bioavailability. The American journal of clinical nutrition. 2002;76(2):447-53. Epub 2002/07/30.

17. Birk Y. The Bowman-Birk inhibitor. Trypsin- and chymotrypsin-inhibitor from soybeans. International journal of peptide and protein research. 1985;25(2):113-31. Epub 1985/02/01.

18. Young D, Ibuki M, Nakamori T, Fan M, Mine Y. Soy-derived di- and tripeptides alleviate colon and ileum inflammation in pigs with dextran sodium sulfate-induced colitis. The Journal of nutrition. 2012;142(2):363-8. Epub 2011/12/23.

19. Sprake EF, Grant VA, Corfe BM. Vitamin D3 as a novel treatment for irritable bowel syndrome: single case leads to critical analysis of patient-centred data. BMJ case reports. 2012;2012. Epub 2012/12/15.

20. Somjen D, Kohen F, Gayer B, Knoll E, Limor R, Baz M, et al. A non-calcemic Vitamin D analog modulates both nuclear and putative membranal estrogen receptors in cultured human vascular smooth muscle cells. The Journal of steroid biochemistry and molecular biology. 2004;89-90(1-5):397-9. Epub 2004/07/01.

21. Moussa L, Bezirard V, Salvador-Cartier C, Bacquie V, Houdeau E, Theodorou V. A new soy germ fermented ingredient displays estrogenic and protease inhibitor activities able to prevent irritable bowel syndrome-like symptoms in stressed female rats. Clinical nutrition (Edinburgh, Scotland). 2013;32(1):51-8. Epub 2012/06/26.

22. Moussa L, Bezirard V, Salvador-Cartier C, Bacquie V, Lencina C, Leveque M, et al. A low dose of fermented soy germ alleviates gut barrier injury, hyperalgesia and faecal protease activity in a rat model of inflammatory bowel disease. PloS one. 2012;7(11):e49547. Epub 2012/11/21.

23. Zender R, Olshansky E. Women's mental health: depression and anxiety. The Nursing clinics of North America. 2009;44(3):355-64. Epub 2009/08/18.

24. Bloedon LT, Jeffcoat AR, Lopaczynski W, Schell MJ, Black TM, Dix KJ, et al. Safety and pharmacokinetics of purified soy isoflavones: single-dose administration to postmenopausal women. The American journal of clinical nutrition. 2002;76(5):1126-37. Epub 2002/10/26.

25. Ullmann U, Metzner J, Frank T, Cohn W, Riegger C. Safety, tolerability, and pharmacokinetics of single ascending doses of synthetic genistein (Bonistein) in healthy volunteers. Advances in therapy. 2005;22(1):65-78. Epub 2005/06/10.

26. Zwart SR, Parsons H, Kimlin M, Innis SM, Locke JP, Smith SM. A 250 mug/week dose of vitamin D was as effective as a 50 mug/d dose in healthy adults, but a regimen of four weekly followed by monthly doses of 1250 mug raised the risk of hypercalciuria. The British journal of nutrition. 2013:1-7. Epub 2013/04/19.
